# Altered gut fungi in systemic lupus erythematosus – A pilot study

**DOI:** 10.3389/fmicb.2022.1031079

**Published:** 2022-12-05

**Authors:** Bao-Zhu Li, Hua Wang, Xian-Bao Li, Qian-Ru Zhang, Rong-Gui Huang, Hong Wu, Yi-Yu Wang, Kai-Di Li, Xiu-Jie Chu, Nv-Wei Cao, Hao-Yue Zhou, Xin-Yu Fang, Rui-Xue Leng, Yin-Guang Fan, Jin-Hui Tao, Zong-Wen Shuai, Dong-Qing Ye

**Affiliations:** ^1^Department of Epidemiology and Biostatistics, School of Public Health, Anhui Medical University, Hefei, Anhui, China; ^2^Inflammatory and Immune Diseases Laboratory of Anhui Province, Hefei, Anhui, China; ^3^The First Hospital of Jiaxing, Jiaxing, Zhejiang, China; ^4^Department of Rheumatology and Immunology, The First Affiliated Hospital of University of Science and Technology of China, Hefei, Anhui, China; ^5^Department of Rheumatology and Immunology, The First Affiliated Hospital of Anhui Medical University, Hefei, Anhui, China

**Keywords:** gut fungi, autoimmune diseases, systemic lupus erythematosus, rheumatoid arthritis, undifferentiated connective tissue diseases, ITS sequencing

## Abstract

**Objective:**

Gut fungi, as symbiosis with the human gastrointestinal tract, may regulate physiology *via* multiple interactions with host cells. The plausible role of fungi in systemic lupus erythematosus (SLE) is far from clear and need to be explored.

**Methods:**

A total of 64 subjects were recruited, including SLE, rheumatoid arthritis (RA), undifferentiated connective tissue diseases (UCTDs) patients and healthy controls (HCs). Fecal samples of subjects were collected. Gut fungi and bacteria were detected by ITS sequencing and 16S rRNA gene sequencing, respectively. Alpha and beta diversities of microbiota were analyzed. Linear discriminant analysis effect size analysis was performed to identify abundance of microbiota in different groups. The correlation network between bacterial and fungal microbiota was analyzed based on Spearman correlation.

**Results:**

Gut fungal diversity and community composition exhibited significant shifts in SLE compared with UCTDs, RA and HCs. Compared with HCs, the alpha and beta diversities of fungal microbiota decreased in SLE patients. According to principal coordinates analysis results, the constitution of fungal microbiota from SLE, RA, UCTDs patients and HCs exhibited distinct differences with a clear separation between fungal microbiota. There was dysbiosis in the compositions of fungal and bacterial microbiota in the SLE patients, compared to HCs. Pezizales, Cantharellales and *Pseudaleuria* were enriched in SLE compared with HCs, RA and UCTDs. There was a complex relationship network between bacterial and fungal microbiota, especially *Candida* which was related to a variety of bacteria.

**Conclusion:**

This study presents a pilot analysis of fungal microbiota with diversity and composition in SLE, and identifies several gut fungi with different abundance patterns taxa among SLE, RA, UCTDs and HCs. Furthermore, the gut bacterial-fungal association network in SLE patients was altered compared with HCs.

## Introduction

Systemic lupus erythematosus (SLE) is characterized by defects in immune tolerance, the excessive production of autoantibodies and the formation of immune complexes (Durcan et al., 2019). The etiology of SLE was thought to arise from the interaction of genetic and environmental factors (Li and Ye, 2011). The contribution of genetic factors to SLE has been examined for more than 40 years. However, researchers believe that only a small portion of the etiology can be explained by these genetic factors. The exact pathogenesis of SLE remains unknown. Studies over the last decade have demonstrated that environmental factors, especially gut microbiota, might play important roles in the development of autoimmune diseases (Mu et al., 2015). The alter of gut microbiota in SLE patients has received increasing attention.

Human intestine contains 10^14^ microorganisms, involving bacteria, archaea, fungi, protozoans, and viruses. Among these, bacteria are the most shining part. With the rapid development of 16S rRNA gene sequencing and metagenomic shotgun sequencing technologies, gut bacterial microbiota in patients with SLE can be studied thoroughly. Several groups worldwide observed both compositional and functional dysbiosis of gut bacterial microbiota in SLE. A study using 16S rRNA gene sequencing demonstrated that *Lactobacillus*, *Prevotella* and *Sneathia* was enriched in SLE patients compared to HCs (Liu et al., 2021). Another study using shotgun sequencing demonstrated that the diversity of gut bacterial microbiota in untreated SLE patients was different compared to healthy controls (HCs), and specific species including *Clostridium species ATCC BaA-442*, *Actinomyces massiliensis*, *Bacteroides fragilis*, and *Clostridium leptum* were enriched in SLE patients (Chen et al., 2021). The above evidence jointly proved that the gut integrity was damaged in SLE patients. In addition, two-sample Mendelian randomization analysis demonstrated a potential causal association between gut bacterial microbiota and the risk of SLE (Xiang et al., 2021). Nevertheless, in addition to gut microbiota, there is a paucity of study looking at the relationships between SLE and other microbial families.

However, previous studies in SLE only focused on gut bacteria microbiota, and the role of fungal microbiota is unclear. The human gastrointestinal tract contains at least 66 fungal genera and 184 fungal species. The gut fungal microbiota is an integral part of gut microbiota ecosystem. Fungi regulate physiology *via* multiple interactions with host cells during symbiosis with the human gastrointestinal tract, despite it their relative abundance is less abundant than bacteria. Recent studies demonstrated that gut fungal microbiota also played an important role in the homeostasis of host immunity (El-Jurdi and Ghannoum, 2017; Iliev and Leonardi, 2017; Drummond and Lionakis, 2019). Ghanoum M has shown that *Candida tropicalis* participated to enhance inflammation by participating in multi-species biofilms in the setting of Crohn’s disease (Hager and Ghannoum, 2017). Spondyloarthritis patients was characterized by higher levels of Ascomycota, and decreased abundance of Basidiomycota (Berthelot et al., 2021). Comprehensive evidence revealed that fungi could elicit T cell responses, from both innate like lymphocytes and adaptive T cells (Dunne et al., 2021). T cells further affected the pathogenesis and treatment of SLE (Sharabi and Tsokos, 2020). Therefore, gut fungal microbiota may be involved in the occurrence and development of SLE.

At present, studies have shown that HCs might not be the only appropriate control choice in the study of autoimmune diseases. Manzano selected rheumatoid arthritis (RA) as the control, and Cano-García chose patients with other autoimmune diseases as controls (Cano-García et al., 2021; Manzano et al., 2021). Interesting founds were observed between autoimmune diseases, including both similar and specific characteristics. As two important kinds of autoimmune diseases (AIDs), the etiologies of SLE and RA are closely related to the impairment of immunity. Undifferentiated connective tissue diseases (UCTDs) are used to define conditions characterized by the presence of signs and symptoms suggestive of a systemic autoimmune disease that do not satisfy the classification criteria for defined connective tissue diseases such as SLE and RA. It is still unknown whether the fungal microbiota is different among UCTDs, RA and SLE. In addition, gut fungal and bacteria microbiota are important components of gut microbiota, and a link between them has been found in several studies (Peng et al., 2020; Manzano et al., 2021). The interaction network between bacterial and fungal microbiota is complex. The destruction of bacteria is a prerequisite for the excessive growth of fungi (Kapitan et al., 2019). It is necessary to study the impact of the relationship between bacteria and fungi on human health and disease.

Therefore, we aim to conduct a pilot study to characterize gut fungal microbiota in SLE patients compared with RA, UCTDs and HCs, and further to analyze the correlation between bacterial and fungal microbiota in SLE patients and HCs. The data will pave the way for the prevention and intervention studies targeting fungal microbiota.

## Materials and methods

### Ethics approval

This study has got the ethical approval from Anhui medical university ethics committee (ethical approval number: 20180079).

### Patients and samples collection

All patients were recruited from the First and Second Affiliated Hospital of Anhui Medical University and provided informed consent. Included SLE patients were newly diagnosed with disease duration <1 year. The diagnosis of SLE was defined according to American College of Rheumatology (ACR)-SLE classification criteria. SLE with the following conditions were excluded: gastrointestinal disease, co-existing autoimmune disease, fever, chronic infectious diseases, severe mental illness, other dermatosis or cancers. All SLE patients have not used antibiotics, probiotics, or synbiotics for at least 1 month prior to enrolment. Finally, nineteen SLE patients were recruited, and sixteen HCs were recruited from the community. The control group did not control dietary activities (such as weight loss) and did not used antibiotics, probiotics, or synbiotics for at least 1 month before recruitment.

To further reveal the fungal characteristics of SLE patients, we also recruited twenty RA patients and nine UCTDs patients from the First and Second Affiliated Hospital of Anhui Medical University. The diagnosis of RA was defined according to the RA classification criteria revised by the American College of Rheumatology. The identification of UCTDs patients was based on the latest classification criteria and judged by rheumatologists (Mosca et al., 2014). None of the participants had a history of other autoimmune diseases, chronic metabolic diseases, chronic infectious diseases, other dermatosis or cancers. Further, none of the participants have used antibiotics, probiotics, or synbiotics for at least 1 month prior to enrolment. Each fecal sample was collected before 8 AM. Whole stools were collected in sterile boxes and immediately homogenized. Per 0.5 g aliquot of samples were frozen at −80°C for further analysis. Informed consents were obtained from all participants involved in this study. A total of sixty-four subjects (nineteen SLE, twenty RA, nine UCTDs and sixteen HCs) were recruited, and fecal samples were collected.

### ITS sequencing

DNA of fecal samples from subjects was extracted by MagPure Soil DNA LQ Kit (Magen, Guangdong, China) for Internal transcribed spacer I (ITS) sequencing. The concentration and integrity of DNA were verified by NanoDrop 2000 spectrophotometer (Thermo Fisher Scientific, Waltham, MA, United States) and agarose gel electrophoresis, respectively. The genome DNA was used as template for PCR amplification with the barcoded primers and Tks Gflex DNA Polymerase (Takara Bio Inc., Kusatsu, Japan). ITS I variable regions were amplified with universal primers ITS 2. After extracting DNA, the obtained fungal genes were amplified using the primers ITS1F (5’-CTTGGTCATTTAGAGGAAGTAA-3′) and ITS2 (5’-GCTGCGTTCTTCATCGATGC-3′; Mukherjee et al., 2014). The process of fungal DNA amplification was shown in Supplementary document. The concentrations of ITS PCR amplicons were then adjusted for sequencing. Sequencing was performed on an Illumina NovaSeq 6,000 with two paired end read cycles of 250 bases each (Illumina Inc., San Diego, CA; OE Biotech Company; Shanghai, China). An optimized and standardized ITS amplicon library preparation protocol (METABIOTE^®^, Genoscreen, Lille, France) was applied.

### 16S rRNA sequencing

DNA of fecal samples from subjects was extracted by QIAamp DNA Stool minikit (Qiagen, Monheim am Rhein, GER) for 16S rRNA sequencing. To prepare gut bacterial microbiome library for sequencing, 16S rRNA were amplified at V3 to V4 hypervariable region by polymerase chain reaction (PCR). For bacterial identification, the V3-V4 regions of the bacterial 16S rRNA genes were amplified from each DNA sample using the 357F (5’-ACTCCTACGGRAGGCAGCAG-3′) and 806R (5’-GGACTACHVGGGTWTCTAAT-3′) primers (Wang et al., 2020). The process of bacterial DNA amplification was shown in Supplementary document. Sequencing was performed on an Illumina NovaSeq 6,000 SP 500 Cycle Reagent Kit (Illumina, San Diego, California, United States).

### Bioinformatics analysis

Original data of ITS with paired-end reads was detected and cut off using Trimmomatic (Version 0.35; Bolger et al., 2014). Clean reads were subjected to primer sequences removal and clustering to generate operational taxonomic units (OTUs) using VSEARCH software with 97% similarity cutoff (Wang et al., 2007). Reads with 75% of bases above Q20 were retained using QIIME software (version 1.8.0; Caporaso et al., 2010). The representative read of each OTU was selected using QIIME package. All representative reads were annotated by BLAST (Altschul et al., 1990) and against UNITE ITS database (utax_reference_dataset_22.08.2016; Kõljalg et al., 2013). The bioinformatics analysis of 16S rRNA were shown in Supplementary document.

### Statistical analysis

The microbial diversity in cecal content samples was estimated using alpha and beta diversities. The beta diversity between gut microbiota were determined by Principal coordinates analysis (PCoA). The Bray-Curtis distance matrix performed by QIIME software was used for PCoA. The Permutational multivariate analysis of variance (PERMANOVA) was used to evaluate the significance of beta diversity. Linear discriminant analysis effect size (LEfSe) analysis was performed to identify abundance of microbiota in different groups. A linear discriminant analysis (LDA) was conducted to estimate the effect size of characteristics with differential abundance. Taxa with *p* < 0.05 or LDA score (log10) ≥4.0 (3.0 in SLE vs. HC) were considered as significantly enriched taxa. Nonparametric factorial Kruskal-Wallis sum-rank test was applied to detect the significance of the difference in four group. The Wilcoxon rank sum test was used to detect the significance of the difference between the two groups. The correlation between fungal and bacterial microbiota was performed using Spearman’s method, and *p* values were corrected. The correlation networks only showed Spearman’s correlation coefficient greater than 0.6. SPSS 23.0 (ver. 23.0; SPSS Inc., Chicago, IL, United States) and R 4.0.3. were used to conduct the tests.

## Results

### Basic characteristics of subjects

Table 1 showed clinical and demographic characteristics of all subjects. All SLE patients had not taken any hormones or medications for treatment (such as hydroxychloroquine, glucocorticoids, etc.) in the last 3 months.

**Table 1:** The clinical and demographic characteristics of all subjects.

	SLE	RA	UCTD	Control
Age, mean ± SD	28.89 ± 8.88	56.65 ± 5.36	44.78 ± 9.52	32.25 ± 9.43
Female, *n* (%)	18 (94.74)	20 (100)	7 (77.78)	16 (100)
BMI, mean ± SD	20.39 ± 2.37	22.78 ± 4.53	22.24 ± 3.48	20.86 ± 2.41
Disease activity, mean ± SD	13.05 ± 5.20	4.08 ± 0.97	–	–
Lupus nephritis, *n* (%)	6 (31.58)	–	–	–
ESR, mm/h, mean ± SD	51.88 ± 25.77	38.80 ± 23.75	40.43 ± 19.68	–
CRP, g/L, mean ± SD	4.55 ± 4.69	21.56 ± 30.43	10.68 ± 22.40	–
C3, g/L, mean ± SD	0.49 ± 0.32	1.21 ± 0.28	0.77 ± 0.22	–
C4, g/L, mean ± SD	0.07 ± 0.07	0.27 ± 0.06	0.26 ± 0.32	–
IgM, g/L, mean ± SD	1.29 ± 0.44	1.37 ± 0.59	1.54 ± 0.50	–
IgG, g/L, mean ± SD	21.67 ± 11.94	11.36 ± 3.06	20.44 ± 7.55	–
IgA, g/L, mean ± SD	3.49 ± 3.61	2.21 ± 1.08	3.06 ± 1.23	–
RF positive, *n* (%)	–	17 (85.00)	3 (33.33)	–
Anti-CCP positive, *n* (%)	–	18 (90.00)	3 (33.33)	–
Anti-Ro/SSA positive, *n* (%)	13 (68.42)	–	6 (66.67)	–
Anti-dsDNA, IU/ml, mean ± SD	433.82 ± 328.01	–	–	–
Anti-Sm positive, *n* (%)	11 (57.89)	–	–	–
Anti-La/SSB positive, *n* (%)	4 (21.05)	–	–	–
Anti-RNP positive, *n* (%)	10 (52.63)	–	–	–
Anti-Ribosome P positive, *n* (%)	9 (47.37)	–	–	–

### Sequencing results

The fungi fraction in the microbiota was analyzed based on ITS sequencing. A total of 4,260,946 clean tags of the sixty-four fecal sample were obtained. After removing the chimera, 4,207,436 valid tags were obtained, with an average of 65,797, 65,556, 63,930 and 66926 valid tags per sample in SLE, RA, UCTDs and HCs, respectively. A total of 4,112 OTUs were obtained after species filtering annotations (Supplementary Figure 1A). The species accumulation curve rose sharply at first. When the number of samples sequenced was 64, the curve showed a gentle trend, indicating that continuing to increase the number of samples sequenced did not significantly increase the number of species (Supplementary Figure 1B).

The bacterial fraction in the microbiota was analyzed based on 16S rRNA gene sequencing. We obtained a total of 1,462,657 high-quality filtered reads after filtering out ambiguous bases, chimeras and homologous single bases, with an average of 38,486 and 45,715 reads per sample in SLE and HCs, respectively. A total of 510 OTUs were obtained after species filtering annotations (Supplementary Figure 1C). The species accumulation curve rose sharply at first, and then began to flatten when the number of samples sequenced was 26, indicating that continuing to increase the number of samples sequenced did not significantly increase the number of species. (Supplementary Figure 1D).

### Altered fungal microbiota in SLE patients compared with RA, UCTD and HCs

**Figure 1:**
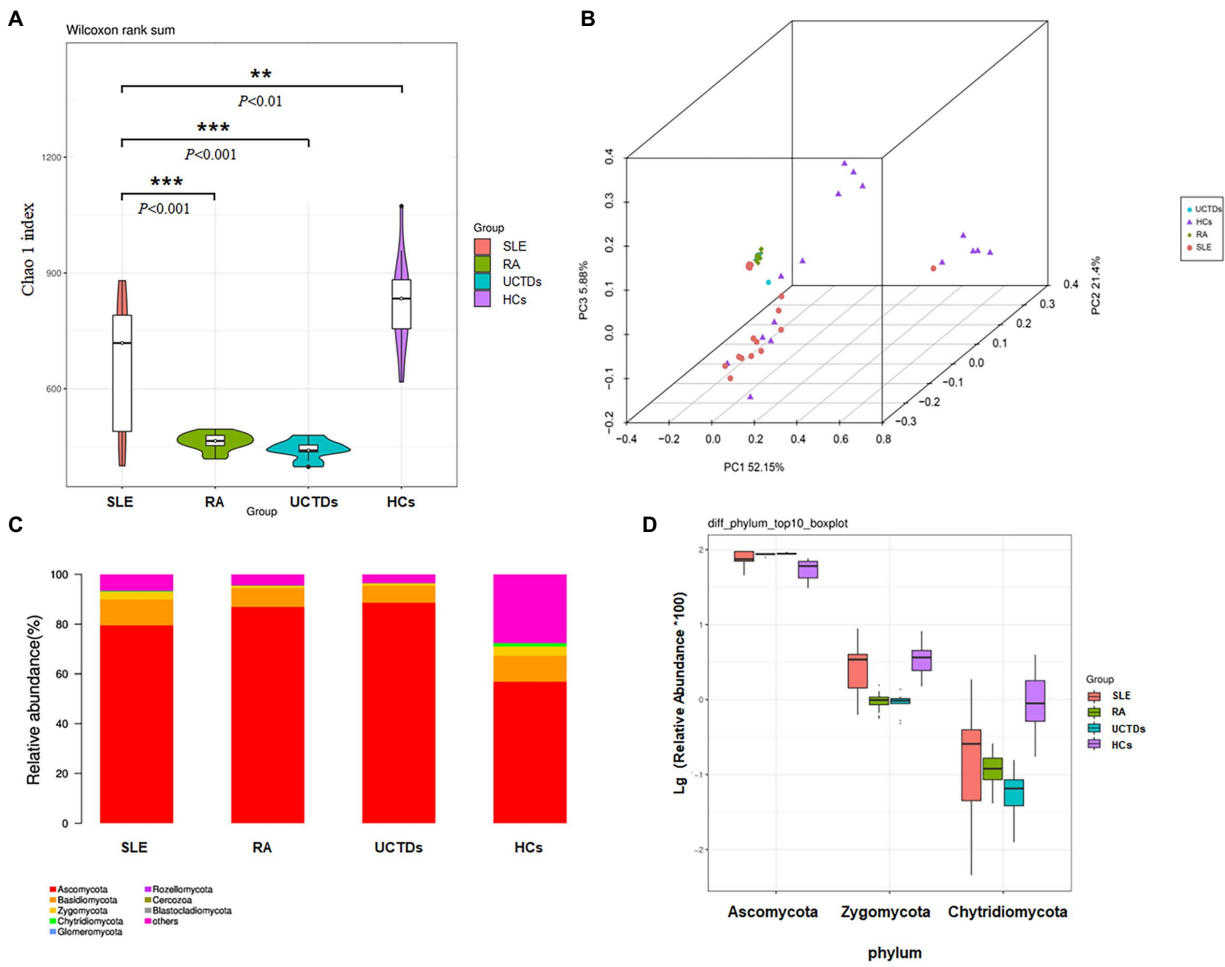
Diversities and relative abundance of gut fungi between SLE, RA, UCTDs and HCs. **(A)** Alpha diversities with Chao 1 index for SLE compared with HCs, RA and UCTDs; **(B)** principal coordinate analysis (PCoA) on Bray-Curtis distance of the fungal microbiota community structures in SLE, HCs, RA and UCTDs; **(C)** the relative abundance of fungi at phyla level of SLE, HCs, RA and UCTDs; **(D)** the relative abundance of fungi at the phylum level differ between SLE, HCs, RA and UCTDs.

We measured alpha diversity of fungal microbiota community in each group using observed species and Chao 1 index to describe species richness and species number, respectively. Alpha diversity was statistically different between SLE patients and HCs. The analysis of alpha diversity, assessed by Chao1 indexes, showed significant difference in SLE patients compared with other three groups (RA, UCTDs and HCs), respectively (Figure 1A). We found that chao1 indexes were significantly decreased in SLE patients compared with HCs, nevertheless increased in SLE patients compared with RA and UCTDs. Further, the observed species were also calculated to analyze the diversity of fungal microbiota in four groups. The results indicated that the number of observed species in SLE patients were far less than that in HCs, whereas no differences were found among SLE, RA, UCTDs patients (Supplementary Figure 2A). Beta diversity was calculated by dividing microbiome variability into major components using PCoA on Bray-Curtis distance to examine the variability of fungal microbiota community in SLE, RA, UCTDs patients and HCs (Figure 1B). The constitution of fungal microbiota from SLE patients and HCs exhibited distinct differences as there was a clear separation between fungal microbiota in two axes (*R*^2^ = 0.2046, *p* = 0.001). PCoA1 and PCoA2 are major two principal axes, which explained 51.3 and 23.25% of variation, respectively (Supplementary Figure 2B). In addition, the beta diversity of SLE patients was different from that of RA or UCTDs (Supplementary Figures 2C,D; *R*^2^ = 0.2808, *p* = 0.001; *R*^2^ = 0.1899, *p* = 0.005, respectively). The fungal community in SLE was separated from other three groups, suggesting that the compositions of gut fungal microbiota from SLE patients were more heterogeneous from others.

The total gut fungal microbiota was revealed through the phylogenetic and taxonomic assessments of the ITS2 region. All fungal populations could be aligned to ten phyla, 204 families and 436 genera. At phylum level, Ascomycota, Basidiomycota and Zygomycota were the three most abundant fungal microbiota in gut (>1% in at least one group) in four groups (Figure 1C). The abundance of Ascomycota was higher in SLE patients compared with HCs, but lower compared with RA and UCTDs (Figure 1C). Although the difference was not statistically significant, we observed that the average ratios of Basidiomycota/Ascomycota in SLE were decreased, compared with HCs (0.146 and 0.172, respectively; Supplementary Figure 3A). The average ratios of Basidiomycota/Ascomycota increased in SLE patients compared with RA and UCTDs patients (0.087 and 0.077, respectively, *p* < 0.05; Supplementary Figure 3A). Besides, significant variation (BH corrected *p* < 0.05) was observed in the abundance of phyla Zygomycota and Chytridiomycota (mean abundance <1%; Figure 1D). Supplementary Tables S1–S3 detailed fungi with significant differences phylum and genus in SLE compared with three groups. It was also noticed that some reads were unclassified at the phylum level, indicating the presence of several unknown fungal taxonomic group. At the genus level, in the fungal microbiota of SLE, the 56 genera that differed compared with HCs (e.g., *Penicillium*; Supplementary Table 1), the 99 genera that differed compared with RA (e.g., *Candida*; Supplementary Table 2), and the 81 genera that differed compared with UCTDs (e.g., *Archaeorhizomyces*; Supplementary Table 3). The top ten different genus were *Zopfiella*, *Staphylotrichum*, *Fusarium*, *Thielavia*, *Chaetomium*, *Humicola*, *Trichoderma*, *Monographella*, *Chaetosphaeria* and *Olpidium* in SLE and HCs (Supplementary Figure 3B). The significant differences of species between four groups were showed in Supplementary Table S4. *Candida* species had increased abundances in SLE than in the RA, UCTDs and HCs group (Supplementary Table S4).

**Figure 2:**
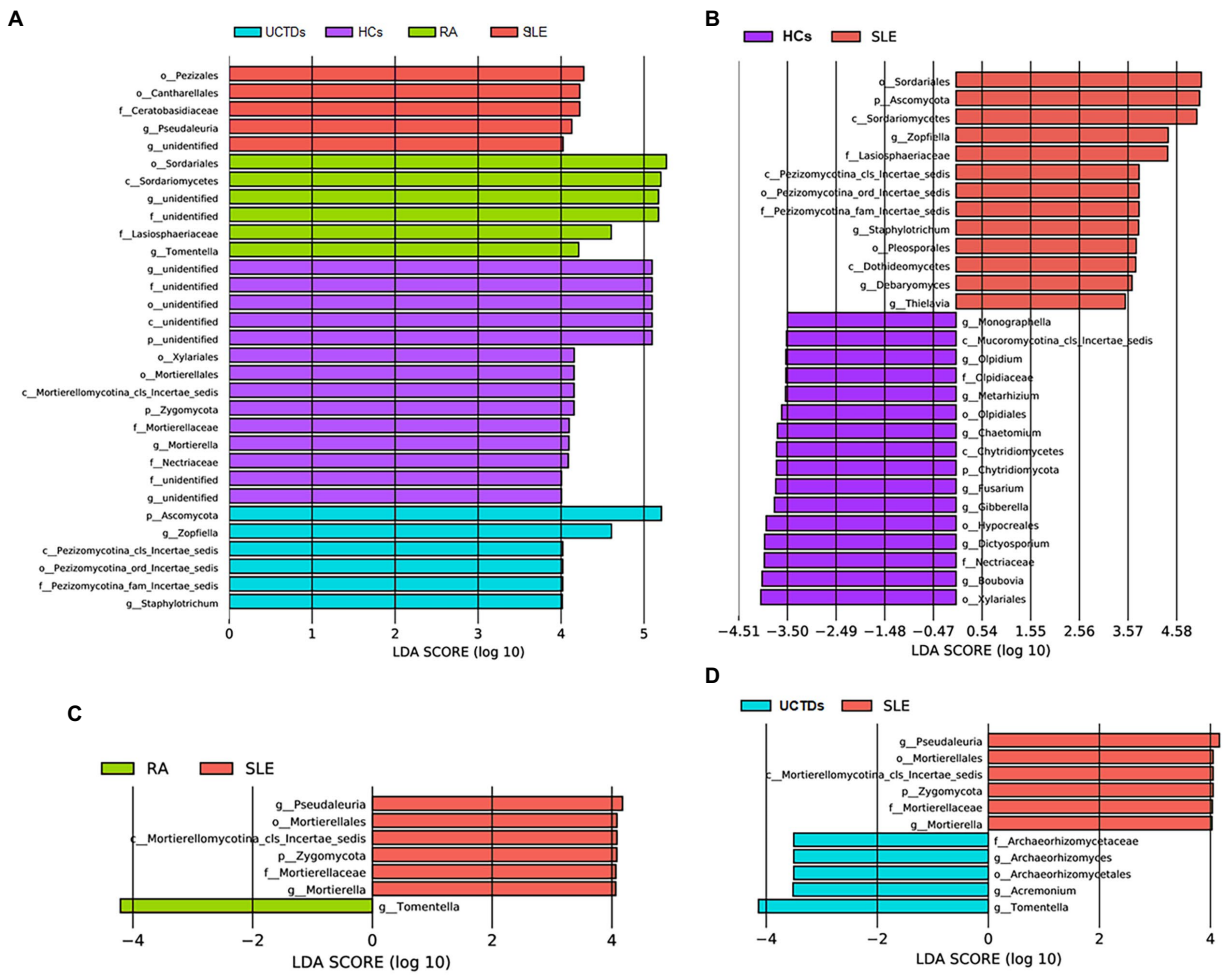
Taxonomic differences in fungal composition between SLE, RA, UCTDs and HCs by LEfSe analysis. **(A)** Taxonomic differences in fungal composition between SLE, HCs, RA and UCTDs; **(B)** taxonomic differences in fungal composition between SLE and HCs; **(C)** taxonomic differences in fungal composition between SLE and RA; **(D)** taxonomic differences in fungal composition between SLE and UCTDs.

To further specifically identify fungal taxa associated with different groups of subjects, we used LEfSe analysis to compare fungal microbiota abundances at different taxonomic level. Pezizales, Cantharellales and *Pseudaleuria* were enriched in SLE compared with HCs, RA and UCTDs (Figure 2A). Interestingly, Zygomycota, Mortierellales, Mortierellaceae and *Mortierella* were enriched in HCs (Figure 2A). Compared with HCs, Ascomycota, *Zopfiella*, *Staphylotrichum*, *Debaryomyces* and *Thielavia* were enriched in SLE patients (Figure 2B). In contrast, Chytridiomycota, *Monographella*, *Olpidium*, *Metarhizium*, *Chaetomium*, *Fusarium*, *Gibberella*, *Dictyosporium* and *Boubovia* were enriched in HCs (Figure 2B). Compared with RA and UCTDs patients, Zygomycota, *Pseudaleuria* and *Mortierella* were significantly more abundant in SLE patients, whereas, *Tomentlla* was the major depleted fungi group in SLE patients (Figures 2C,D). In addition, *Archaeorhizomyces* and *Acremonium* were enriched in UCTDs patients compared with SLE patients (Figure 2D).

We further analyzed the correlation between different fungi and clinical indicators (Supplementary Figure 3C). We found Mortierellales, *Mortierella* and *Fusarium*, which enriched in HCs were negatively related to the SLEDAI, positively related to Complement 4. *Tomentella* enriched in RA, was negatively related to the SLEDAI. Archaeorhizomyces, Archaeorhizomycetaceae and *Archaeorhizomyces*, which enriched in UCTDs were negatively related to the SLEDAI. Ascomycota was negatively correlated with C4 and lgG. Chytridiomycota, Olpidiaceae, Hypocreales, Mortierellales, *Fusarium*, *Monographella*, *Olpidium* and *Pseudaleuria* showed a positive correlation with lgG. We found no direct correlation between differential fungi and Anti-dsDNA, lgA, lgM, ESR and C3.

### Altered bacterial microbiota in SLE patients compared with HCs

**Figure 3:**
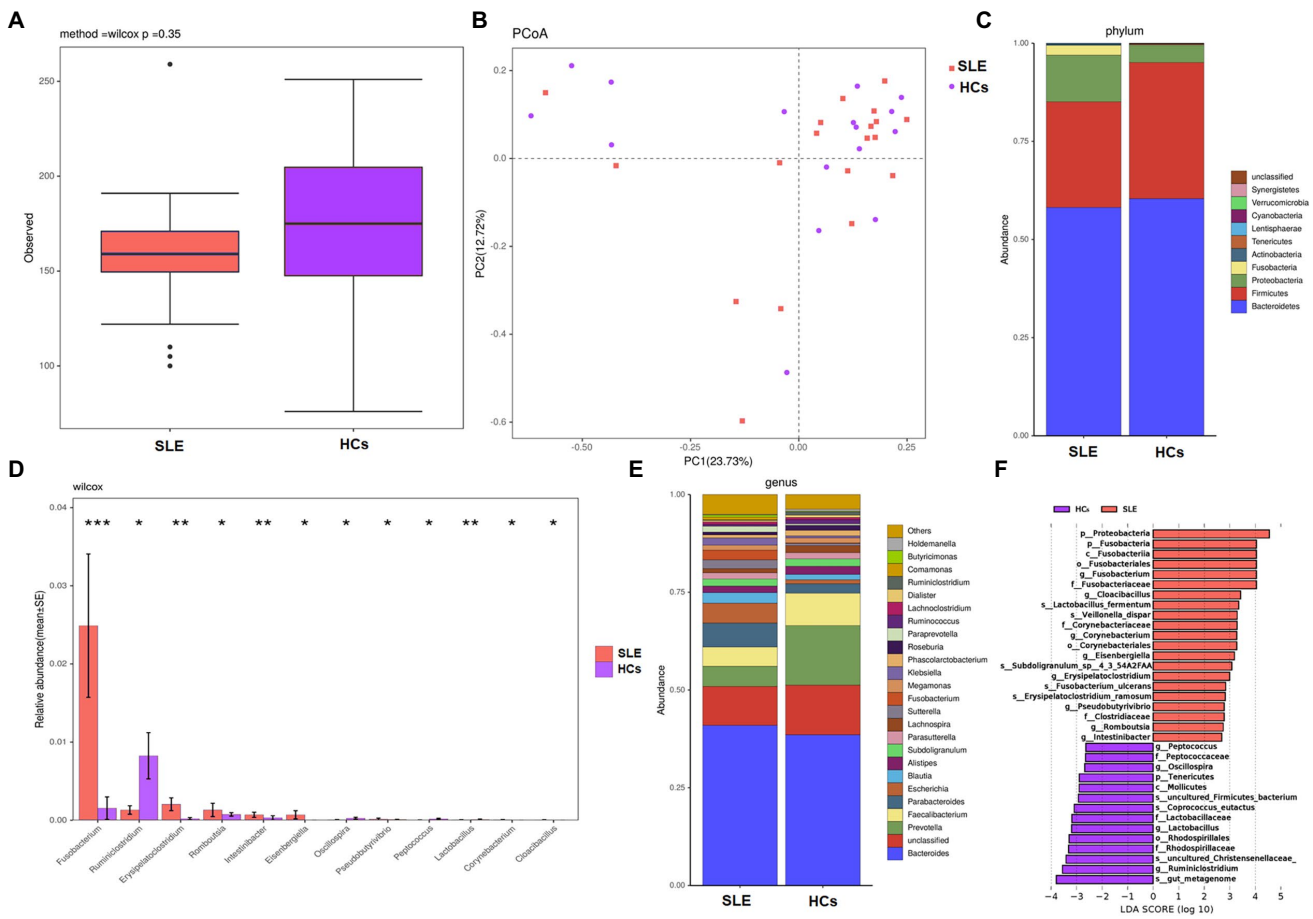
Diversities and compositions of gut bacteria between SLE and HCs. **(A)** Alpha diversities with observed species for SLE compared with HCs; **(B)** principal coordinate analysis (PCoA) on Bray-Curtis distance of the bacterial microbiota community structures in SLE and HCs; **(C)** the relative abundance of bacteria at phyla level of SLE and HCs; **(D)** the relative abundances of SLE and HCs at genus level were among the top 12 differences. **(E)** The relative abundance of bacteria at genus level of SLE and HCs; **(F)** taxonomic differences in bacterial composition between SLE and HCs by LEfSe analysis.

We measured the alpha diversity of bacterial microbiota community between two groups using observed species and Shannon index. The alpha diversity (observed species and Shannon index) of bacterial in SLE patients tended to decrease compared with HCs (Figure 3A; Supplementary Figure 4), whereas no statistical differences were found. Beta diversity was calculated by dividing microbiome variability into major components using PCoA on Bray-Curtis distance to examine the variability of bacterial microbiota community among SLE patients and HCs. PCoA1 and PCoA2 are major two principal axes, which explained 23.73 and 12.72% of variation, respectively (Figure 3B).

The analysis of bacterial community composition indicated that Bacteroidetes, Firmicutes, Proteobacteria, Fusobacteria and Actinobacteria were the dominant phyla in both SLE patients and HCs (Figure 3C). At the phylum level, the abundance of Proteobacteria and Fusobacteria were increased and the abundance of Firmicutes was found decreased in SLE patients compared with HCs. The average ratio of Firmicutes/Bacteroidetes in SLE was decreased, compared with HCs (0.46 and 0.57, respectively). At the genus level *Fusobacterium*, *Erysipelatoclostridium*, *Romboutsia*, *Intestinibacter* and *Eisenbergiella* were increased in relative abundance, whereas *Ruminiclostridium* was decreased in relative abundance in SLE compared with HCs (*p* < 0.05; Figure 3D). Furthermore, we noticed that *Prevotella* and *Faecalibacterium* were deficient, whereas *Escherichia* and *Parabacteroides* were enriched in SLE patients compared with HCs (Figure 3E).

To further specifically identify bacterial taxa associated with different groups of subjects, we used LEfSe analysis to compare bacterial microbiota abundances at different taxonomic level and LDA to estimate the effect size of each feature. As shown in Figure 3F, the comparison between SLE and HCs groups revealed that the major enriched bacterial group in SLE patients were Proteobacteria and Fusobacteria phylum, especially the *Fusobacterium* genus. At the genus level, *Romboutsia*, *Pseudobutyrivibrio*, *Erysipelatoclostridium*, and *Intestinibacter* were enriched in SLE patients, whereas *Lactobacillus*, *Oscillospira* and *Ruminiclostridium* were more abundant in HCs.

### Altered bacterial-fungal associations in SLE patients compared with HCs

**Figure 4:**
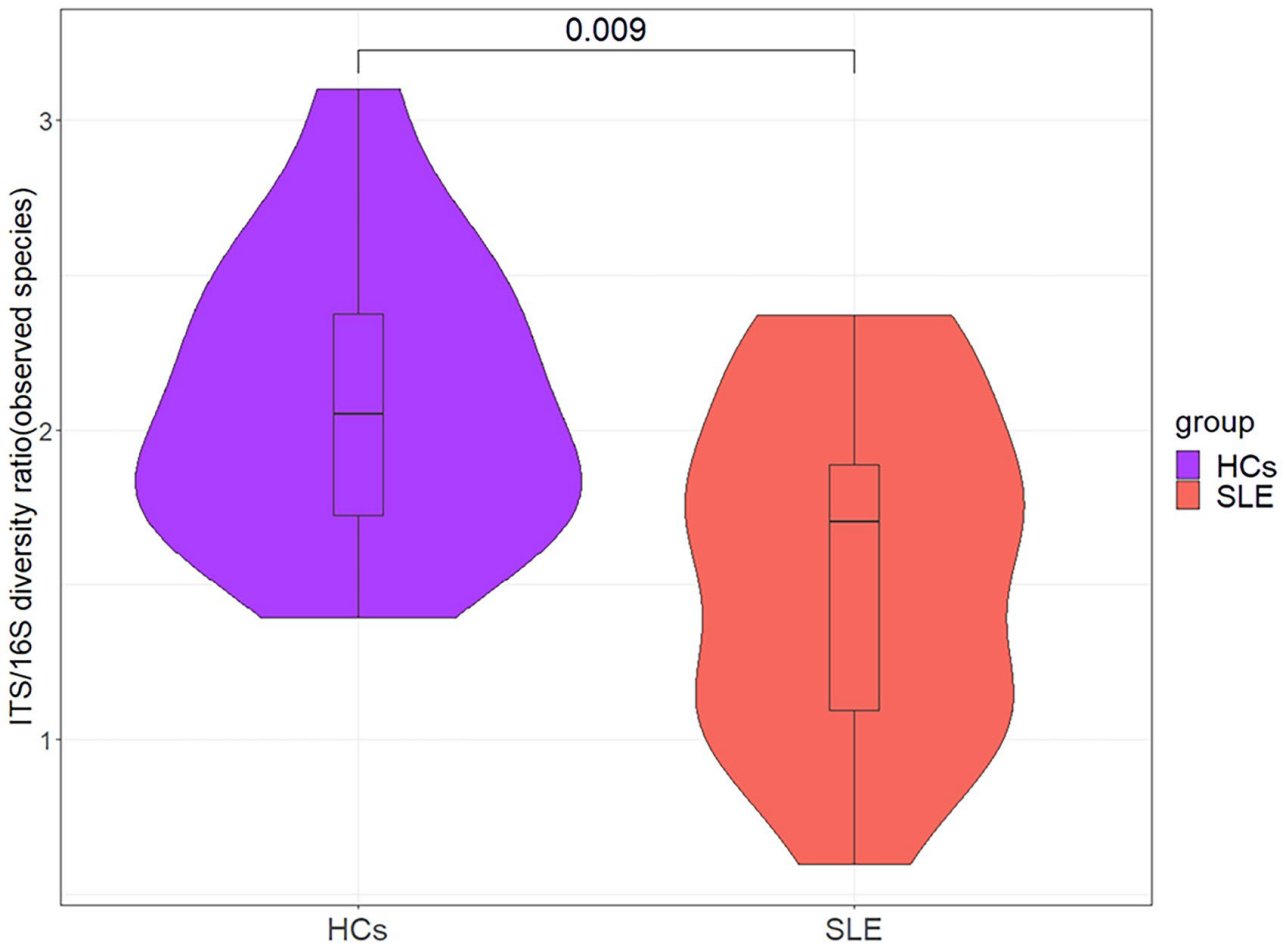
The ITS/16S diversity ratio between SLE and HCs at the genus level.

**Figure 5:**
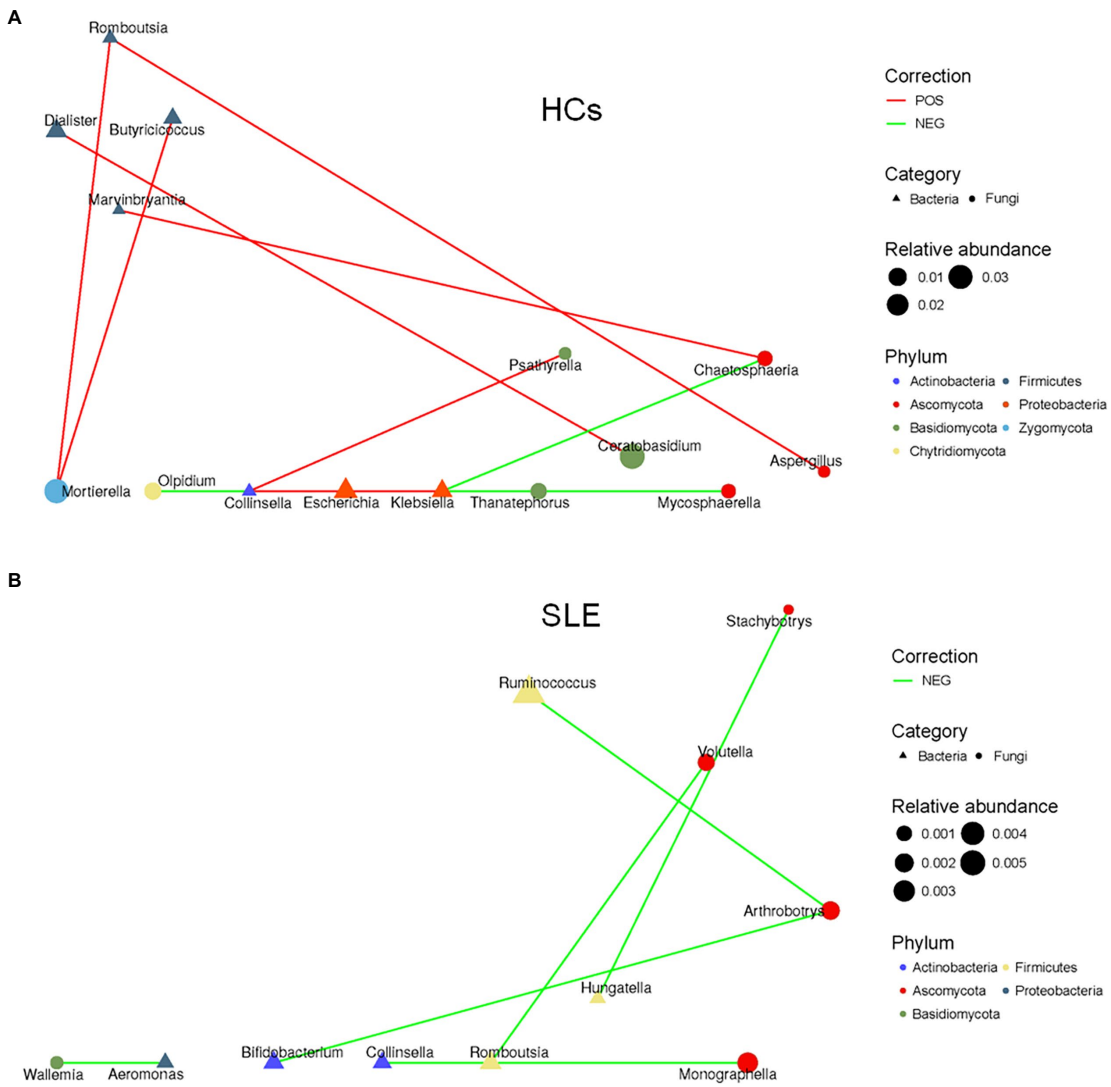
Altered bacterial-fungal associations between SLE and HCs. **(A)** The bacterial-fungal association network at the genus level in HCs; **(B)** the bacterial-fungal association network at the genus level in SLE.

To explore the balance of bacterial and fungal diversity in the gut, ITS/16S was performed based on observed species between SLE patients and HCs. Compared with HCs, the ITS/16S ratio was significantly decreased in SLE patients (*p* < 0.05; Figure 4). This suggested that the balance of bacteria and fungi was disrupted in SLE patients compared with HCs. To test this hypothesis, we analyzed the correlation between gut fungi and bacteria at the genus level. We found a positive correlation between *Aspergillus* and *Anaerostipes* in SLE (Supplementary Figure 5A). Interestingly, *Candida* was negatively correlated with *Prevotella*, *Parasutterella* and *Ruminiclostridium*, nevertheless positively correlated with *Aeromonas* in SLE*, which* differed from that of the HCs (Supplementary Figures 5A,B). We further analyzed the bacterial-fungal association network at the genus level to explore the potential roles of fungi and bacteria. The results indicated a richer bacterial and fungal association network in HCs (Figures 5A,B). Interestingly, we found a negative correlation between fungal and bacterial abundance in bacterial and fungal association network in SLE patients. For example, *Monographella* was negatively correlated with *Collinsella*, *Arthrobotrys* was negatively correlated with *Ruminococcus* and *Bifidobacterium* in SLE patients, and *Volutella* was also negatively correlated with *Romboutsia*. Taken together, these results suggested a complex relationship between gut fungal and bacterial microbiota.

## Discussion

Recently, gut microbiota has become one of the major and hot topics of studies on SLE, and extensive evidence suggested that gut microbiota might affect SLE by regulating immune functions. Most gut microbiota studies were focused on bacteria, with few studies reporting fecal fungi in SLE. In fact, fungi are an integral part of human gut microbiota and have impacts on human health, beneficially or harmfully. Furthermore, bacterial and fungal microbiota co-exist in human gut, forming a complex network of interactions.

This study has several interesting findings. First, the gut fungal diversity and community composition exhibited significant shifts in SLE patients compared with HCs. Besides, the gut fungal diversity of SLE patients was different from that of RA and UCTDs patients. Second, there was dysbiosis in the composition of fungal microbiota in SLE patients. Our results supported the mucosal origins hypothesis theory that inflammation and autoimmunity might have relationship with gut fungi. Thirdly, we further confirmed changes in gut bacteria in SLE patients compared with HCs, which agrees with previous studies (Kim et al., 2019; Pan et al., 2021). Besides, at the genus level, we also found altered fungal and bacterial interaction networks, compared to HCs. There was a complex network of relationships between gut fungal and bacterial microbiota. Thus, larger cohorts are required in future to verify our findings.

Recently, there was a view that HCs might not be the most appropriate control for autoimmune disease studies. Li et al. investigated the characteristics of gut bacteria microbiota in SLE patients compared with RA patients and HCs (Li et al., 2019b). UCTDs patients have also been used as a comparison group for SLE in another study (Kaufman et al., 2021). Therefore, different types of control selection help us to further understand the characteristics of gut fungal microbiota in SLE patients. Other AIDs (such as multiple sclerosis and Sjögren’s syndrome) could be considered as options for control to explore SLE characteristics in future studies. However, since RA tends to occur in middle-aged and elderly people, the age of RA patients in our study was not matched with SLE patients (Lee and Weinblatt, 2001; Scott et al., 2010). In the future, more studies are needed to consider matching the ages of SLE patients with other AIDs to verify our results.

Compared with gut bacterial microbiota, the biodiversity of fungi was lower and more uneven. The unevenness led to great distances among subjects, which we could see from Figure 1B of PcoA analysis. Most samples in our study were dominated by two or three fungal phyla. The phyla Ascomycota and Basidiomycota were mainly fungal microbiome in HCs of this study, in which the former was particularly predominant. These observations were compatible with those of Japanese and Western healthy participants (Sokol et al., 2017; Imai et al., 2019). Ascomycota is one of the dominant phyla of fungi kingdom. It plays an essential role in the physiology, growth, and metabolism of host species and produces some crucial enzymes that aid in the digestion of complex carbohydrates (Köhler et al., 2017). The abundances of Ascomycota were different in SLE, RA, UCTDs patients and HCs, indicating the dysbiosis of fungal microbiota might involve in multiple ways of pathogenesis of SLE. Moreover, Ascomycota and Basidiomycota abundances correlated negatively with each other. Basidiomycota to Ascomycota ratios showed decreased trends in SLE, RA, and UCTDs, indicating that this ratio might represent a useful fungal dysbiosis index.

Notably, in this study, we found some differential fungi enriched in SLE, RA, and UCTDs. In patient groups, Pezizales, Cantharellales, and *Pseudaleuria* were enriched in SLE patients, whereas *Tomentella* and Archaeorhizomyces were enriched in RA and UCTDs patients, respectively. The characteristic fungi in these diseases might be potential fungal markers which could be used to distinguish SLE, RA and UCTDs. Especially, the positive correlation between Archaeorhizomyces and CD4^+^ T-cell count was found in immune-related diseases (Chang et al., 2021). Although changes in the structure and composition of gut fungi in SLE patients were found in our results compared with RA, CUTDs, and HCs, the relationship of gut fungi with SLE and the biological functions of fungi are still unclear. In a word, future studies with larger sample sizes are urgent to explore the role and biological functions of fungi as markers in SLE and other AIDs.

At genera level, this study found *Mortierella*, belonging to phylum Zygomycota, produces large amounts gamma-linoleic acid (GLA), which are poly unsaturated fatty acids involved in suppressing inflammation (Sergeant et al., 2016; Jayasudha et al., 2018). It was supposed that GLA may have effect of anti-inflammation. A previous study using animal model indicated that GLA-supplemented diets could alleviate the level of inflammation (Fan and Chapkin, 1998). Moreover, in a double-blind randomized controlled trial, evidence showed that supplementing GLA could change the fatty acid composition of peripheral blood mononuclear cell phospholipids, so as to inhibit lymphocyte proliferation (Sergeant et al., 2016). GLA-derived metabolites or the competitive inhibition with arachidonic acid in the synthesis of pro-inflammatory arachidonic acid product might induce the anti-inflammation of GLA (Thies et al., 2001).

Additionally, *Candida* species had increased abundances in the SLE group than other groups in this study. At present, the prevalence of invasive mould is gradually increasing in patients receiving large doses of corticosteroids, in which *Candida* species was one kind of major species. *Candida parapsilosis,* presenting an increased trend in patients in this study (Supplementary Table S4), is a fungus which could producing prostaglandins from exogenous arachidonic acid (Chakraborty et al., 2019). In SLE, the synthesis of Prostaglandin D_2_ (PGD_2_) increased in SLE patients and induced the aggregation of CXC chemokine receptor 4 through the PGD2 - PGD2 receptors axis, making contributions in the occurrence and development of SLE (Pellefigues et al., 2018). Meanwhile, recent evidence indicated prostaglandins might mediate the occurrence of chronic inflammation by promoting differentiation of Th1 and Th17 cells (Yao and Narumiya, 2019). The specific relationship between *Candida* species and SLE needs to be further explored.

Fungi are eukaryotes, but share some similarities with mammalian cells. Which is different is the presence of a cell wall provides the fungus with protection from environmental stresses. Fungal cell wall is mainly composed of B-glucans (polymers of glucose), chitin (polymer of N-acetylglucosamine) and mannans (Gow et al., 2017). Previous evidence demonstrated that fungal cell wall provoked the innate immune system of phagocytes *via* pattern recognition receptors (PRRs; Hatinguais et al., 2020). It was produced by immune system cells including Toll-like receptors (TLRs), C-type lectin like receptors (CLRs) and Nod-like receptors (NLRs; Hatinguais et al., 2020). PRRs mediated innate immunity through recognizing fungus-associated molecular patterns in fungal cell walls. Besides, three classical, lectin and alternative pathways were three major complement activation pathways and acted as PRRs performing function (Speth et al., 2008). Dectin-1 is a C-type lectin like receptor (CLRs). Upregulation of dectin-1 could activate NF-kB and NFAT in innate cells, leading to increased pro-inflammatory cytokines. It has also been proved that surface molecules of fungi stimulate the toll-like receptors (TLRs), TLR2 and TLR4, thus provoking pro-inflammatory responses (Bartemes and Kita, 2018). Although it remains unclear whether fungal dysbiosis observed in AIDs patients is associated with its immunological and pro-inflammatory properties, this pilot study provided some clues leading researchers to notice the fungus causing disease. And due to the lack of function database, it is unable to analyze the function of gut fungi in four groups, so that the relationship between fungal function and human health is still unclear. Further studies on functional changes of the fungal microbiome are needed using a metagenomics approach.

Bacterial and fungal microbiota live together in gut, and they will inevitably interact with each other (Kapitan et al., 2019; Pareek et al., 2019). The change of ITS/16S diversity ratio reflects the change of fungus-bacterial microbiota balance. A recent study showed that gut fungal and bacterial microbiota are interdependent, especially the interaction of gut bacteria microbiota with *Candida albicans* (Pérez, 2021). Another study in ankylosing spondylitis also found that *Candida* was associated with a variety of bacteria (Li et al., 2019a). In our study, *Candida* was found to be negatively correlated with *Prevotella*, *Parasutterella* and *Ruminiclostridium*, and positively correlated with *Aeromonas*. A study found that *Candida tropicalis* can interact with bacteria to affect the formation of biofilms, which may be involved in inflammatory processes (Hager et al., 2019). However, due to the cross-sectional study, it is difficult to deeply explore the relationship between bacteria and fungi in depth. In the future, prospective studies are needed to explore the association between fungi and bacteria in SLE patients.

## Conclusion

In summary, this study presents a pilot analysis of fungal microbiota diversity and composition in SLE, and the correlation between gut fungal and bacteria microbiota. Both diversity and composition of fungal microbiota are imbalanced in SLE compared with RA, UCTDs and HCs. Thus, fungi might serve as a useful diagnostic biomarker and therapeutic target. In addition, the gut bacterial-fungal association network in SLE patients was altered compared with HCs. Further studies on gut fungi and their related functions are urgently needed to establish any causality in disease pathogenesis.

## Data availability statement

The datasets presented in this study can be found in online repositories. The names of the repository/repositories and accession number(s) can be found below: NCBI - (BioProject ID: PRJNA877434 http://www.ncbi.nlm.nih.gov/bioproject/877434).

## Ethics statement

The studies involving human participants were reviewed and approved by Anhui medical university ethics committee (ethical approval number: 20180079). The patients/participants provided their written informed consent to participate in this study.

## Author contributions

B-ZL contributed to conception and design of the study and wrote the first draft of the manuscript. HuW collected data and revised draft. X-BL collected data, performed the statistical analysis and revised draft. Q-RZ collected data, provided interpretation and revised draft. R-GH, HoW, and K-DL collected data and revised draft. Y-YW and X-JC collected data and performed the statistical analysis. N-WC and H-YZ collected data and revised draft. X-YF contributed to the interpretation of the study. R-XL and Y-GF performed the statistical analysis. J-HT and Z-WS collected data. D-QY contributed to conception and design of the study. All authors contributed to manuscript revision, read, and approved the submitted version.

## Funding

This study was supported by National Natural Science Foundation of China (81803310 and 82073652), the Peak Discipline of Public Health and Preventive Medicine, Anhui Medical University, the Research Fund of Anhui Institute of translational medicine (2021zhyx-C21), Undergraduate Innovation and Entrepreneurship Training Program in Anhui Province, and the Grants for Scientific Research of BSKY from Anhui Medical University (XJ201619).

## Conflict of interest

The authors declare that the research was conducted in the absence of any commercial or financial relationships that could be construed as a potential conflict of interest.

## Publisher’s note

All claims expressed in this article are solely those of the authors and do not necessarily represent those of their affiliated organizations, or those of the publisher, the editors and the reviewers. Any product that may be evaluated in this article, or claim that may be made by its manufacturer, is not guaranteed or endorsed by the publisher.
