# Supplementary material for: Altered gut fungi in systemic lupus erythematosus – A pilot study
Source: Front Microbiol. 2022 Dec 5;13:1031079. doi: 10.3389/fmicb.2022.1031079 (PMC9760866; doi:10.3389/fmicb.2022.1031079)
Supplement: Supplementary file 1 [file Data_Sheet_1.docx]

**Supplementary methods**

**The process of fungal DNA amplification in ITS sequencing**

For the first amplification, a 30-μl-volume system was prepared and mixed well, containing 15μl of Gflex PCR Buffer, 1μl of primer F, 1 μl of primer R, 50ng of template DNA, 0.6μl of Tks Gflex DNA Polymerase and remaining volume of ddH_2_O. Then the mixture was amplified under following cycling conditions: denaturation at 94°C for 5 min, 26 cycles of amplification at three temperatures (30s at 94 °C, 30s at 56°C and 20s at 72°C), extension 72°C for 5min. Amplicon quality of the PCR products was visualized using gel electrophoresis. Then the PCR products were purified with Agencourt AMPure XP beads (Beckman Coulter Co., USA). For the second amplification, another mixture with a 30-μl-volume system was prepared, containing 15μl of Gflex PCR Buffer, 1μl of Adapter I5, 1 μl of Adapter I7, 50 ng of template DNA, 0.6 μl of Tks Gflex DNA Polymerase and remaining volume of ddH_2_O. Then the mixture was reacted at 94°C for 5 min, followed by 7 cycles of 94 °C for 30s, 56°C for 30s and 72°C for 20s, and extended at 72°C for 5min. The second-round PCR products were quantified using Qubit dsDNA assay kit after being purified.

**16S sequencing study**

***Extraction and determination of bacteria***

QIAamp DNA Stool Mini Kit (Qiagen, Monheim am Rhein, GER) was used to extract DNA of stool from subjects. To prepare stool microbiome library for sequencing, 16S rRNA were amplified at V3 to V4 hypervariable region by quantitative polymerase chain reaction (qPCR). And two-way sequencing was performed, and primers were designed using the forward primer (5'-ACTCCTACGGRAGGCAGCAG-3’) and reverse primer (5'-GGACTACHVGGGTWTCTAAT-3'). The two-step qPCR amplification method was used build a library. The first-step qPCR amplification was performed employing a step cycling protocol consisting of 94 °C for 2min, 25 cycles of 94 °C for 30s, 56°C for 30s, and 72 °C for 30s, ending with the final elongation at 72 °C for 5 min and 10°C heat preservation. All qPCR products were recovered using the AxyPrepDNA gel recovery kit, and quantified using FTC-3000TM real-time qPCR instrument (Shanghai Fengling Biological Technology Co.,Ltd. China). Then subjected to secondary qPCR amplification, adding the adapters required for sequencing. The second-step qPCR amplification was performed employing a step cycling protocol consisting of 94 °C for 2min, 8 cycles of 94 °C for 30s, 56 °C for 30s, and 72 °C for 30s, ending with the final elongation at 72 °C for 5 min and 10°C heat preservation. Then, qPCR products were recycled using AxyPrepDNA gel recovery kit (AXYGEN, U.S.A.).

***Statistical analysis***

For the sequencing of 16S rRNA gene-based amplicons, the amplicon library was prepared using a Novaseq 6000 SP 500 Cycle Reagent Kit (Illumina, San Diego, California, USA). Low-quality, ambiguous reads or homologous, including mismatching reads, and raw reads shorter than 50bp, were firstly filtered for the following assembly. The maximum allowable error ratio of an overlap region is 0.2. According to overlap relationship between the paired-end reads, pairs of reads were merged into a sequence and the minimum overlap length is 10bp. Trimmomatic (Version 0.35) was used to control sequence quality. Paired-end clean reads were further merged using FLASH (Version 1.2.11).

Perform operational taxonomic units (OTUs) clustering on the Clean Tags, and then complete OTU species classification by annotating OTU. Use USEARCH (Version V8.1.1756) to cluster the assembled sequences into OUT. The representative sequence of OTU was obtained by UPARSE software clustering under 97% similarity. The chimera generated by qPCR amplification was removed from the OTU representative sequence by UCHIME software. The USEARCH_GLOBAL method was used to align all the sequences back to the OTU representative sequences, and the statistical table of the abundance of each sample in each OTU was obtained. Next, species annotation is performed by comparing the OTU representative sequence with the database (Silva128) through mothur (classifie.seqs, Version 1.33.3) software, and the confidence threshold is set to 0.6.

The species richness and evenness of microbial communities reflected by α-diversity. The differences in diversity of species between samples were analyzed by β-diversity. The alpha-diversity and beta-diversity were analyzed using mothur (Version 1.33.3). The significance of α-diversity and β-diversity was determined by the Wilcoxon rank sum test. linear discriminant analysis effect size (LEfSe) analysis was used to identify the significant enrichment of microorganisms in different groups. We detected the characteristics with significant abundance difference and found the taxa with significant abundance difference by nonparametric factorial Kruskal-Wallis (KW) sum-rank test, followed by a Linear Discriminant Analysis (LDA) to estimate the effect size of each microbial characteristics with differential abundance. P value < 0.05 (Kruskal -- Wallis test) and LDA score (log10) greater than 2.0 were considered as significantly enriched taxa. Furthermore, based on Kyoto Encyclopedia of Genes and Genomes (KEGG) database and KEGG orthologs (KOs), phylogenetic investigation of communities by reconstruction of unobserved states (PICRUSt) analysis was conducted to predict possible metabolic pathways of gut microbial in SLE patients and HCs. Calypso was used to identify featured function with linear discriminant analysis effect size.

**Supplementary Tables**

**Supplementary Table 1.** The detailed fungi with significant differences in SLE compared with HCs

| Taxon | HCs_mean | SLE_mean | *P*-value |
| --- | --- | --- | --- |
| Ascomycota | 0.570531343 | 0.793574071 | 0.000999001 |
| unidentified | 0.272937786 | 0.066558593 | 0.000999001 |
| Chytridiomycota | 0.01328741 | 0.003262452 | 0.000999001 |
| Blastocladiomycota | 2.27E-05 | 0 | 0.046953047 |
| Thielavia | 0.008141 | 0.014588 | 0.000999 |
| Trichoderma | 0.003613 | 0.007159 | 0.000999 |
| Olpidium | 0.00777 | 0.001421 | 0.000999 |
| Penicillium | 0.002089 | 0.003496 | 0.000999 |
| Preussia | 0.004186 | 0.001109 | 0.000999 |
| Taeniolella | 0.001099 | 0.002471 | 0.000999 |
| Arthrobotrys | 0.000855 | 0.001957 | 0.000999 |
| Cercophora | 0.001064 | 0.000188 | 0.000999 |
| Rhodotorula | 0.000456 | 0.000125 | 0.000999 |
| Ophiosphaerella | 0.000162 | 7.19E-05 | 0.000999 |
| Cyphellophora | 0.00014 | 6.36E-06 | 0.000999 |
| Staphylotrichum | 0.016052 | 0.028449 | 0.001998 |
| Fusarium | 0.022949 | 0.011132 | 0.001998 |
| Chaetomium | 0.014392 | 0.004575 | 0.001998 |
| Monographella | 0.008822 | 0.002773 | 0.001998 |
| Zopfiella | 0.069774 | 0.122481 | 0.002997 |
| Vermispora | 0.000208 | 7.02E-05 | 0.002997 |
| Neonectria | 9.08E-05 | 4.83E-05 | 0.002997 |
| Trichocladium | 8.09E-05 | 9.54E-06 | 0.002997 |
| Cladorrhinum | 0.000292 | 0.000101 | 0.003996 |
| Verticillium | 6.18E-05 | 2.53E-05 | 0.003996 |
| Peziza | 0.004094 | 0.001431 | 0.005994 |
| Bipolaris | 0.000189 | 7.77E-05 | 0.006993 |
| Modicella | 2.79E-05 | 0 | 0.007992 |
| Dendryphion | 8.92E-05 | 8.07E-07 | 0.008991 |
| Volutella | 0.000784 | 0.001471 | 0.00999 |
| Geomyces | 4.11E-05 | 1.61E-06 | 0.012987 |
| Graphostroma | 1.92E-05 | 0 | 0.013986 |
| Leptodontidium | 5.45E-05 | 1.10E-05 | 0.015984 |
| Dictyosporium | 2.54E-05 | 0 | 0.017982 |
| Lachnella | 8.72E-05 | 1.26E-05 | 0.018981 |
| Humicola | 0.005952 | 0.008373 | 0.021978 |
| Gliocladiopsis | 0.000254 | 0.000136 | 0.022977 |
| unidentified | 0.687511 | 0.633759 | 0.023976 |
| Pyrenochaetopsis | 0.000675 | 0.000366 | 0.023976 |
|  |  |  |  |
| Taxon | HCs_mean | SLE_mean | *P*-value |
| Debaryomyces | 0 | 0.00022 | 0.023976 |
| Stachybotrys | 0.000716 | 0.000444 | 0.024975 |
| Gibberella | 8.22E-06 | 0 | 0.025974 |
| Boubovia | 3.41E-05 | 0 | 0.027972 |
| Apodus | 0.000231 | 9.65E-05 | 0.028971 |
| Powellomyces | 4.31E-05 | 8.00E-07 | 0.028971 |
| Mycosphaerella | 0.003304 | 0.001653 | 0.02997 |
| Coprinellus | 0.001106 | 0.001359 | 0.033966 |
| Chrysosporium | 8.89E-05 | 2.52E-05 | 0.033966 |
| Metarhizium | 2.24E-05 | 7.66E-07 | 0.035964 |
| Acremonium | 0.001374 | 0.000925 | 0.036963 |
| Genolevuria | 0 | 3.18E-06 | 0.036963 |
| Xylaria | 2.03E-05 | 0 | 0.042957 |
| Stilbella | 8.33E-06 | 0 | 0.042957 |
| Cortinarius | 0.001518 | 0.002081 | 0.043956 |
| Meyerozyma | 1.34E-05 | 3.21E-06 | 0.043956 |
| Chaetosphaeria | 0.004706 | 0.004257 | 0.044955 |
| Lophiostoma | 5.24E-05 | 2.32E-05 | 0.045954 |
| Paraglomus | 1.41E-05 | 0 | 0.046953 |
| Erythricium | 1.46E-05 | 0 | 0.047952 |
| Blumeria | 4.66E-05 | 1.04E-05 | 0.048951 |

**Supplementary Table 2.** The detailed fungi with significant differences in SLE compared with RA

| Taxon | RA_mean | SLE_mean | *P*-value |
| --- | --- | --- | --- |
| Zygomycota | 0.009691 | 0.031082 | 0.000999 |
| Tomentella | 0.034032 | 0.001515 | 0.000999 |
| Mortierella | 0.006216 | 0.026747 | 0.000999 |
| Pseudaleuria | 0.001969 | 0.028538 | 0.000999 |
| Archaeorhizomyces | 0.009489 | 0.00049 | 0.000999 |
| Acremonium | 0.007802 | 0.000925 | 0.000999 |
| Taeniolella | 0.004352 | 0.002471 | 0.000999 |
| Chaetomium | 0.002035 | 0.004575 | 0.000999 |
| Corynascus | 0.000176 | 0.002272 | 0.000999 |
| Guehomyces | 0.000275 | 0.001949 | 0.000999 |
| Zymoseptoria | 0.00176 | 0 | 0.000999 |
| Mycosphaerella | 0.000125 | 0.001653 | 0.000999 |
| Psathyrella | 1.59E-05 | 0.00147 | 0.000999 |
| Subulicystidium | 0.001231 | 4.54E-05 | 0.000999 |
| Parasola | 0.001142 | 1.11E-05 | 0.000999 |
| Serendipita | 0.001026 | 0 | 0.000999 |
| Metarhizium | 0.00094 | 7.66E-07 | 0.000999 |
| Papiliotrema | 0.000834 | 9.50E-05 | 0.000999 |
| Inocybe | 0.000761 | 9.98E-05 | 0.000999 |
| Sporobolomyces | 0.000536 | 0.000142 | 0.000999 |
| Tuber | 0.000638 | 0 | 0.000999 |
| Exophiala | 0.000121 | 0.000506 | 0.000999 |
| Trichophyton | 0.000591 | 7.54E-06 | 0.000999 |
| Geoglossum | 0.000557 | 0 | 0.000999 |
| Fusidium | 0.000419 | 0 | 0.000999 |
| Geopora | 0.000315 | 7.96E-06 | 0.000999 |
| Pluteus | 0.000291 | 2.80E-05 | 0.000999 |
| Leptodontidium | 0.000269 | 1.10E-05 | 0.000999 |
| Lecythophora | 1.83E-05 | 0.000254 | 0.000999 |
| Solicoccozyma | 0 | 0.000224 | 0.000999 |
| Paurocotylis | 0.000212 | 0 | 0.000999 |
| Hannaella | 4.07E-05 | 0.000153 | 0.000999 |
| Cristinia | 0.00016 | 0 | 0.000999 |
| Moleospora | 0 | 0.00013 | 0.000999 |
| Oidiodendron | 0 | 9.82E-05 | 0.000999 |
| Funneliformis | 0 | 4.11E-05 | 0.000999 |
| Simocybe | 6.15E-05 | 0.000834 | 0.001998 |
| Marasmiellus | 0.000304 | 1.10E-05 | 0.001998 |
| Glomus | 0.000243 | 1.11E-05 | 0.001998 |
| Myrmecridium | 0.000197 | 0 | 0.001998 |
| Myrothecium | 2.60E-05 | 0.000144 | 0.001998 |
| Taxon | RA_mean | SLE_mean | *P*-value |
| Bipolaris | 0 | 7.77E-05 | 0.001998 |
| Endosporium | 0 | 2.81E-05 | 0.001998 |
| Kondoa | 0 | 1.61E-05 | 0.001998 |
| Alogomyces | 8.16E-06 | 0.00019 | 0.002997 |
| Gliocladiopsis | 3.70E-05 | 0.000136 | 0.002997 |
| Colletotrichum | 0 | 4.86E-05 | 0.002997 |
| Thermomyces | 0 | 3.75E-05 | 0.003996 |
| Chaetosphaeria | 0.000186 | 0.004257 | 0.004995 |
| Bolbitius | 7.00E-05 | 0.002696 | 0.004995 |
| Coprinellus | 0.001156 | 0.001359 | 0.004995 |
| Apiotrichum | 3.99E-05 | 0.00017 | 0.004995 |
| Dimorphospora | 0.000156 | 0 | 0.004995 |
| Yamadazyma | 0 | 5.95E-05 | 0.005994 |
| Eremiomyces | 0.000142 | 0 | 0.006993 |
| Lectera | 6.35E-05 | 0.000877 | 0.007992 |
| Bullera | 0 | 2.94E-05 | 0.007992 |
| Laetisaria | 0 | 7.26E-05 | 0.008991 |
| Podospora | 0.001762 | 0.001169 | 0.00999 |
| Pisolithus | 7.56E-05 | 0.000835 | 0.00999 |
| Trapelia | 0 | 5.21E-05 | 0.010989 |
| Udeniomyces | 0 | 2.05E-05 | 0.010989 |
| Limnoperdon | 0.000162 | 0 | 0.011988 |
| Cylindrocladiella | 0 | 4.55E-05 | 0.011988 |
| Conocybe | 0.000281 | 0.000848 | 0.012987 |
| Articulospora | 0 | 4.52E-05 | 0.013986 |
| Melanocarpus | 1.53E-05 | 0.000105 | 0.018981 |
| Hebeloma | 9.06E-05 | 0 | 0.01998 |
| Thielavia | 0.017697 | 0.014588 | 0.021978 |
| Phlyctochytrium | 0 | 4.03E-05 | 0.021978 |
| Stagonospora | 8.98E-05 | 0 | 0.026973 |
| Cutaneotrichosporon | 0 | 4.02E-05 | 0.026973 |
| Pyrenochaetopsis | 0.000892 | 0.000366 | 0.027972 |
| Neonectria | 0 | 4.83E-05 | 0.027972 |
| Didymosphaeria | 1.21E-05 | 0 | 0.027972 |
| Calyptella | 7.54E-05 | 0.000215 | 0.028971 |
| Phialophora | 0 | 6.94E-05 | 0.030969 |
| Lachnella | 0 | 1.26E-05 | 0.031968 |
| Thanatephorus | 0.000721 | 0.002443 | 0.032967 |
| Preussia | 0.000367 | 0.001109 | 0.032967 |
| Microascus | 0 | 5.97E-05 | 0.032967 |
| Trichoderma | 0.008798 | 0.007159 | 0.033966 |
| Aureobasidium | 0 | 4.78E-06 | 0.034965 |
| Clavaria | 0.000141 | 1.15E-05 | 0.036963 |
| Taxon | RA_mean | SLE_mean | *P*-value |
| Xylaria | 6.61E-05 | 0 | 0.036963 |
| Cladosporium | 9.14E-05 | 0.000185 | 0.037962 |
| Trechispora | 4.22E-05 | 8.67E-05 | 0.037962 |
| Remersonia | 0 | 4.11E-05 | 0.038961 |
| Cystolepiota | 0.000118 | 0 | 0.03996 |
| Vermispora | 3.25E-05 | 7.02E-05 | 0.03996 |
| Chrysosporium | 0 | 2.52E-05 | 0.040959 |
| Entoloma | 6.93E-06 | 0.000154 | 0.042957 |
| Herpotrichia | 0 | 1.02E-05 | 0.042957 |
| Candida | 0.003511 | 0.008798 | 0.043956 |
| Phaeotheca | 0 | 5.78E-05 | 0.044955 |
| Hypoxylon | 4.88E-05 | 0 | 0.045954 |
| Cercosporella | 0 | 2.34E-05 | 0.045954 |
| Pyrenochaeta | 0 | 2.43E-06 | 0.045954 |
| Alternaria | 4.54E-05 | 6.02E-05 | 0.047952 |
| Monascus | 0 | 0.000224 | 0.04995 |

**Supplementary Table 3**. The detailed fungi with significant differences in SLE compared with UCTDs

| Taxon | UCTDs_mean | SLE_mean | *P*-value |
| --- | --- | --- | --- |
| Zygomycota | 0.00924 | 0.031082 | 0.000999 |
| Other | 0 | 9.77E-05 | 0.020979 |
| Mortierella | 0.005909 | 0.026747 | 0.000999 |
| Tomentella | 0.02867 | 0.001515 | 0.000999 |
| Acremonium | 0.007407 | 0.000925 | 0.000999 |
| Chaetosphaeria | 7.85E-05 | 0.004257 | 0.000999 |
| Archaeorhizomyces | 0.006998 | 0.00049 | 0.000999 |
| Guehomyces | 0.000163 | 0.001949 | 0.000999 |
| Zymoseptoria | 0.001943 | 0 | 0.000999 |
| Subulicystidium | 0.00127 | 4.54E-05 | 0.000999 |
| Stachybotrys | 0 | 0.000444 | 0.000999 |
| Parasola | 0.000897 | 1.11E-05 | 0.000999 |
| Metarhizium | 0.000809 | 7.66E-07 | 0.000999 |
| Fusidium | 0.0007 | 0 | 0.000999 |
| Serendipita | 0.000587 | 0 | 0.000999 |
| Tuber | 0.000543 | 0 | 0.000999 |
| Geoglossum | 0.000521 | 0 | 0.000999 |
| Clavaria | 0.000489 | 1.15E-05 | 0.000999 |
| Trichophyton | 0.000487 | 7.54E-06 | 0.000999 |
| Pluteus | 0.000412 | 2.80E-05 | 0.000999 |
| Cladosporium | 0 | 0.000185 | 0.000999 |
| Paurocotylis | 0.000349 | 0 | 0.000999 |
| Phaeoacremonium | 2.90E-05 | 0.000141 | 0.000999 |
| Leptodontidium | 0.000291 | 1.10E-05 | 0.000999 |
| Myrmecridium | 0.000258 | 0 | 0.000999 |
| Calyptella | 0 | 0.000215 | 0.001998 |
| Custingophora | 3.69E-05 | 0.000388 | 0.002997 |
| Exophiala | 7.54E-05 | 0.000506 | 0.003996 |
| Cristinia | 0.00028 | 0 | 0.003996 |
| Corynascus | 0.000139 | 0.002272 | 0.004995 |
| Cortinarius | 5.32E-05 | 0.002081 | 0.004995 |
| Pseudaleuria | 0.001795 | 0.028538 | 0.005994 |
| Solicoccozyma | 0 | 0.000224 | 0.005994 |
| Simocybe | 2.18E-05 | 0.000834 | 0.006993 |
| Eremiomyces | 0.000111 | 0 | 0.008991 |
| Funneliformis | 0 | 4.11E-05 | 0.008991 |
| Papiliotrema | 0.000722 | 9.50E-05 | 0.00999 |
| Itersonilia | 0.000372 | 7.62E-05 | 0.010989 |
| Alogomyces | 0 | 0.00019 | 0.012987 |
| Mycosphaerella | 8.75E-05 | 0.001653 | 0.013986 |
| Pisolithus | 9.68E-05 | 0.000835 | 0.013986 |
| Moleospora | 0 | 0.00013 | 0.014985 |
| Taxon | UCTDs_mean | SLE_mean | *P*-value |
| Apiotrichum | 2.58E-05 | 0.00017 | 0.016983 |
| Geopora | 0.000265 | 7.96E-06 | 0.016983 |
| Kondoa | 0 | 1.61E-05 | 0.016983 |
| Pyrenochaetopsis | 0.000833 | 0.000366 | 0.017982 |
| Inocybe | 0.000632 | 9.98E-05 | 0.018981 |
| Conocybe | 0.000256 | 0.000848 | 0.01998 |
| Bipolaris | 0 | 7.77E-05 | 0.01998 |
| Septoria | 5.92E-05 | 0 | 0.01998 |
| Sphaerulina | 5.06E-05 | 0 | 0.01998 |
| Limnoperdon | 0.00013 | 0 | 0.020979 |
| Dictyosporium | 6.28E-05 | 0 | 0.020979 |
| Endosporium | 0 | 2.81E-05 | 0.020979 |
| Bolbitius | 7.66E-05 | 0.002696 | 0.021978 |
| Marasmiellus | 0.000213 | 1.10E-05 | 0.021978 |
| Preussia | 0.00032 | 0.001109 | 0.022977 |
| Myxocephala | 9.04E-05 | 0.000237 | 0.022977 |
| Cystolepiota | 6.95E-05 | 0 | 0.022977 |
| Hypoxylon | 4.43E-05 | 0 | 0.022977 |
| Leptoxyphium | 0.000188 | 0 | 0.023976 |
| Other | 0 | 9.77E-05 | 0.024975 |
| Hasegawazyma | 6.10E-05 | 0 | 0.025974 |
| Trichothecium | 3.79E-05 | 0 | 0.026973 |
| Lecythophora | 4.87E-05 | 0.000254 | 0.027972 |
| Nectria | 9.15E-05 | 0 | 0.028971 |
| Sporobolomyces | 0.000654 | 0.000142 | 0.02997 |
| Melanophyllum | 5.77E-05 | 0 | 0.02997 |
| Thermomyces | 0 | 3.75E-05 | 0.030969 |
| Leucoagaricus | 1.48E-05 | 0 | 0.031968 |
| Ascobolus | 0 | 6.97E-05 | 0.033966 |
| Psathyrella | 0.000111 | 0.00147 | 0.034965 |
| Mrakia | 0.000141 | 8.00E-07 | 0.035964 |
| Geosmithia | 4.38E-05 | 0 | 0.038961 |
| Conlarium | 1.74E-05 | 0.000253 | 0.041958 |
| Arthrographis | 0 | 5.41E-05 | 0.041958 |
| Yamadazyma | 0 | 5.95E-05 | 0.042957 |
| Zygopleurage | 0 | 6.64E-05 | 0.043956 |
| Trapelia | 0 | 5.21E-05 | 0.043956 |
| Apodus | 0 | 9.65E-05 | 0.044955 |
| Laetisaria | 0 | 7.26E-05 | 0.045954 |
| Bulleribasidium | 7.54E-05 | 0 | 0.048951 |
| Paecilomyces | 0 | 9.41E-05 | 0.04995 |

**Supplementary Table 4 Fungi with significant differences at species level between four groups**

| Taxon | UCTDs_mean | RA_mean | HCs_mean | SLE_mean | *P*-value |
| --- | --- | --- | --- | --- | --- |
| Tomentella_galzinii | 0.028627581 | 0.033847467 | 0 | 0 | 2.18E-12 |
| Onygenales_sp | 0.003466614 | 0.004360768 | 1.89E-05 | 1.27E-05 | 1.14E-11 |
| Acremonium_persicinum | 0.006539886 | 0.006809772 | 4.92E-05 | 6.35E-05 | 9.73E-11 |
| Zymoseptoria_brevis | 0.001942949 | 0.001759998 | 6.73E-06 | 0 | 1.17E-10 |
| Geoglossum_umbratile | 0.00052096 | 0.000557325 | 0 | 0 | 2.17E-10 |
| Archaeorhizomyces_sp | 0.006996336 | 0.009432629 | 0.000407563 | 0.000488078 | 2.24E-10 |
| Tuber_taiyuanense | 0.000543237 | 0.000627732 | 0 | 0 | 8.75E-10 |
| Parasola_lilatincta | 0.000577763 | 0.000733468 | 9.23E-06 | 0 | 1.13E-09 |
| Metarhizium_marquandii | 0.000589437 | 0.000639373 | 7.42E-06 | 0 | 1.38E-09 |
| Mortierella_sp | 0.00246066 | 0.002231922 | 0.024126897 | 0.021836775 | 1.40E-09 |
| Inocybe_adaequata | 0.000604306 | 0.000578597 | 0 | 0 | 3.16E-09 |
| Trichophyton_rubrum | 0.000486507 | 0.000590871 | 5.77E-06 | 7.54E-06 | 4.39E-09 |
| Psathyrellaceae_sp | 0.001907056 | 0.002249543 | 0.00015509 | 0.000307063 | 6.70E-09 |
| Paurocotylis_pila | 0.000348671 | 0.000211651 | 0 | 0 | 1.08E-08 |
| Subulicystidium_perlongisporum | 0.001269715 | 0.001231277 | 0.000196982 | 4.54E-05 | 1.94E-08 |
| Serendipita_sp | 0.000587359 | 0.001025651 | 0 | 0 | 2.02E-08 |
| Sebacinaceae_sp | 0.00121992 | 0.001212244 | 0.000111184 | 8.39E-05 | 3.17E-08 |
| Sporobolomyces_clavatus | 0.000531215 | 0.000381848 | 5.66E-06 | 5.62E-06 | 4.51E-08 |
| Taeniolella_phialosperma | 0.003276089 | 0.004352284 | 0.001099067 | 0.002470949 | 7.21E-08 |
| Fusidium_griseum | 0.00070004 | 0.000418676 | 0 | 0 | 7.49E-08 |
| Exophiala_pisciphila | 2.48E-05 | 0 | 0.000228476 | 0.000385541 | 1.49E-07 |
| Bipolaris_maydis | 0 | 0 | 0.000188984 | 7.77E-05 | 1.65E-07 |
| Metarhizium_anisopliae | 0.000219591 | 0.000300247 | 0 | 7.66E-07 | 2.15E-07 |
| Coprinellus_verrucispermus | 0.000582215 | 0.000635737 | 0 | 0 | 2.38E-07 |
| Conocybe_striaepes | 0 | 0 | 0.000368389 | 0.000427735 | 3.01E-07 |
| Nectriaceae_sp | 0.001480719 | 0.001424985 | 0.012434134 | 0.004245946 | 3.32E-07 |
| Rhodotorula_ferulica | 0 | 0 | 0.000407356 | 0.000113843 | 3.98E-07 |
| Agaricomycetes_sp | 0.000785737 | 0.001206594 | 0.003108221 | 0.000142418 | 6.28E-07 |
| Chaetomium_grande | 0.000558511 | 0.000581375 | 0.013383674 | 0.003032612 | 7.47E-07 |
| Corynascus_sp | 0.000138601 | 0.000176465 | 0.002161059 | 0.00227244 | 7.81E-07 |
| Cladorrhinum_flexuosum | 0 | 0 | 0.000246693 | 9.45E-05 | 7.93E-07 |
| Montagnulaceae_sp | 0.00038169 | 0.000554537 | 0 | 0 | 9.09E-07 |
| Podospora_cochleariformis | 0.000582419 | 0.000516505 | 0 | 0 | 9.19E-07 |
| Basidiomycota_sp | 0.000182202 | 0.000160529 | 0.004364734 | 0.001912234 | 9.92E-07 |
| Tremellomycetes_sp | 0.000426048 | 0.000370784 | 0.002342916 | 0.001291308 | 1.04E-06 |
| Preussia_terricola | 0.000128243 | 0.000201453 | 0.003143619 | 0.000850948 | 1.33E-06 |
| Papiliotrema_fuscus | 0.000423636 | 0.00054337 | 0 | 5.41E-06 | 1.47E-06 |
| Mycosphaerella_tassiana | 8.75E-05 | 0.000125173 | 0.003304479 | 0.001652696 | 2.00E-06 |
| Stachybotrys_microspora | 0 | 3.65E-05 | 0.000607371 | 0.00034863 | 2.08E-06 |
| Trichoderma_harzianum | 0.00429258 | 0.005501425 | 0.002001103 | 0.004424842 | 2.13E-06 |
| Sordariales_sp | 0.509787915 | 0.525382325 | 0.23169067 | 0.434276162 | 2.61E-06 |
| Tomentella_ellisii | 0 | 6.36E-05 | 0.00061165 | 0.000683779 | 2.72E-06 |
| Ophiosphaerella_sp | 0 | 0 | 0.000136611 | 7.19E-05 | 2.75E-06 |
| Olpidiales_sp | 6.64E-05 | 0.000121647 | 0.002089081 | 0.000644975 | 3.27E-06 |
| Thielavia_sp | 0.017290279 | 0.017670847 | 0.008067735 | 0.014276805 | 3.42E-06 |
| Chaetosphaeria_raciborskii | 7.85E-05 | 0.000185978 | 0.004677491 | 0.004134335 | 3.67E-06 |
| Zopfiella_marina | 0.148889351 | 0.148807524 | 0.069034029 | 0.122281464 | 4.20E-06 |
| Bolbitius_coprophilus | 7.66E-05 | 4.52E-05 | 0.003359116 | 0.002695163 | 4.38E-06 |
| Aspergillus_fumigatus | 0 | 0 | 1.02E-05 | 7.17E-05 | 4.80E-06 |
| Guehomyces_pullulans | 0.000163218 | 0.000275253 | 0.002607561 | 0.001949489 | 5.05E-06 |
| Xylariales_sp | 0.002238363 | 0.001944469 | 0.022742102 | 0.005562111 | 5.12E-06 |
| Acaulosporaceae_sp | 0 | 1.21E-05 | 0.000129325 | 6.58E-05 | 6.28E-06 |
| Parasola_kuehneri | 0.000319405 | 0.000408306 | 0 | 0 | 6.83E-06 |
| Peziza_buxea | 2.85E-05 | 5.14E-05 | 0.002621201 | 0.001095589 | 7.51E-06 |
| Mortierella_hyalina | 0.000120947 | 3.08E-05 | 0.00081635 | 0.000892101 | 8.76E-06 |
| Sporobolomyces_roseus | 3.99E-05 | 0 | 8.47E-05 | 3.33E-05 | 9.03E-06 |
| Cercophora_samala | 0.00010244 | 2.18E-05 | 0.000668241 | 0.000165326 | 9.26E-06 |
| Olpidium_brassicae | 0.000304224 | 0.000541982 | 0.007770109 | 0.001421411 | 9.59E-06 |
| Monographella_cucumerina | 0.000686762 | 0.000716895 | 0.008810527 | 0.00277268 | 1.23E-05 |
| Humicola_phialophoroides | 0.009103472 | 0.008154499 | 0.00404574 | 0.007044579 | 1.39E-05 |
| Humicola_nigrescens | 0.000281213 | 0.00023331 | 0.001905663 | 0.001311346 | 1.40E-05 |
| Psathyrella_sp | 0 | 6.72E-06 | 0.000293443 | 0.000258115 | 1.52E-05 |
| Aspergillus_terreus | 0.000482007 | 0.000255549 | 2.32E-05 | 3.84E-05 | 1.63E-05 |
| Thermomyces_lanuginosus | 0 | 0 | 5.74E-05 | 3.75E-05 | 2.50E-05 |
| Pezizales_sp | 0.000219215 | 0.000323409 | 0.004702714 | 0.004651523 | 2.53E-05 |
| Conocybe_apala | 4.38E-05 | 4.45E-05 | 0.00020468 | 0.000117171 | 2.67E-05 |
| Gliocladiopsis_curvata | 4.72E-05 | 3.70E-05 | 0.000253861 | 0.000136326 | 2.69E-05 |
| Fungi_sp | 0.035201854 | 0.043735293 | 0.272901058 | 0.066521524 | 2.97E-05 |
| Halosphaeriaceae_sp | 0.001274039 | 0.001261812 | 0.001098694 | 0.000214126 | 3.17E-05 |
| Chaetomiaceae_sp | 0.002244358 | 0.00351906 | 0.008790028 | 0.008074225 | 3.47E-05 |
| Coprinopsis_pachyderma | 4.07E-05 | 0.000104057 | 0.001657017 | 0.000482765 | 4.20E-05 |
| Conocybe_anthracophila | 0 | 7.61E-06 | 0.00015925 | 0.000231907 | 4.27E-05 |
| Simocybe_sp | 2.18E-05 | 6.15E-05 | 0.000870267 | 0.000834468 | 4.69E-05 |
| Geopora_sp | 0.000264765 | 0.00028562 | 0 | 7.96E-06 | 5.07E-05 |
| Vermispora_spermatophaga | 3.42E-05 | 3.25E-05 | 0.000207696 | 7.02E-05 | 5.12E-05 |
| Pezizomycetes_sp | 0.000578362 | 0.000813046 | 3.55E-05 | 0.00013589 | 5.20E-05 |
| Preussia_sp | 0.000192062 | 0.00011911 | 0.000983825 | 0.000238339 | 5.25E-05 |
| Zopfiella_sp | 7.55E-05 | 4.88E-05 | 0.000730299 | 0.000150277 | 5.43E-05 |
| Funneliformis_caledonium | 0 | 0 | 6.21E-05 | 3.21E-05 | 5.43E-05 |
| Alogomyces_tanneri | 0 | 8.16E-06 | 0.000108513 | 0.000189619 | 5.46E-05 |
| Verticillium_albo-atrum | 0 | 0 | 6.00E-05 | 2.53E-05 | 6.03E-05 |
| Moleospora_sp | 0 | 0 | 0.000236434 | 0.000130333 | 6.36E-05 |
| Sordariaceae_sp | 7.97E-05 | 1.79E-05 | 8.16E-05 | 7.93E-07 | 6.65E-05 |
| Staphylotrichum_coccosporum | 0.037261315 | 0.031151507 | 0.016014503 | 0.028432288 | 9.59E-05 |
| Conocybe_pubescens | 4.19E-05 | 0 | 6.65E-05 | 2.30E-05 | 0.000105574 |
| Myrothecium_cinctum | 3.10E-05 | 2.60E-05 | 0.000210996 | 0.000143611 | 0.000106149 |
| Psathyrella_pygmaea | 0.000110735 | 0 | 0.000774665 | 0.001159521 | 0.00011085 |
| Leptodontidium_sp | 0.000290953 | 0.000269491 | 5.45E-05 | 1.10E-05 | 0.000129496 |
| Inocybe_curvipes | 0 | 3.32E-05 | 7.73E-05 | 9.98E-05 | 0.000134629 |
| Lachnella_villosa | 0 | 0 | 8.25E-05 | 1.18E-05 | 0.00013572 |
| Cladosporium_grevilleae | 0 | 4.01E-05 | 0.00033191 | 0.000101416 | 0.000142539 |
| Cladorrhinum_bulbillosum | 0 | 0 | 4.55E-05 | 6.09E-06 | 0.000145135 |
| Sordariomycetes_sp | 0.00526313 | 0.004586789 | 0.012275447 | 0.004281885 | 0.000147775 |
| Lecythophora_fasciculata | 4.87E-05 | 1.83E-05 | 0.000314301 | 0.000254215 | 0.000151325 |
| Leotiomycetes_sp | 2.53E-05 | 0.00010145 | 0.000493592 | 0.000253089 | 0.000153133 |
| Arthrobotrys_microscaphoides | 0.002345902 | 0.002124961 | 0.000795924 | 0.001886146 | 0.000195644 |
| Dimorphospora_sp | 0 | 0.000155793 | 0 | 0 | 0.000197296 |
| Funneliformis_mosseae | 0 | 0 | 4.45E-05 | 6.31E-06 | 0.000206905 |
| Dendryphion_nanum | 0 | 0 | 8.92E-05 | 8.07E-07 | 0.000218593 |
| Fusarium_sp | 0.003251782 | 0.005304302 | 0.014876779 | 0.004144311 | 0.000239475 |
| Calyptella_capula | 0 | 7.54E-05 | 0.000591797 | 0.000214769 | 0.000246833 |
| Orbiliaceae_sp | 0.003366042 | 0.002979899 | 0.001325619 | 0.00261632 | 0.000248637 |
| Saccharomycetales_sp | 0.000553267 | 0.000382053 | 8.11E-05 | 9.40E-05 | 0.000253729 |
| Cladorrhinum_sp | 0.000257629 | 0.000244862 | 0 | 0 | 0.0002646 |
| Thelephoraceae_sp | 0.00206084 | 0.00188821 | 0.020776335 | 0.022782188 | 0.000264734 |
| Acremonium_nepalense | 0.000209848 | 7.40E-05 | 0 | 0 | 0.000265523 |
| Rhodotorula_fragaria | 5.12E-06 | 7.58E-05 | 0 | 0 | 0.000275085 |
| Neonectria_sp | 6.37E-05 | 0 | 7.93E-05 | 4.83E-05 | 0.000285094 |
| Microascales_sp | 0.000298282 | 0.000154862 | 0.000764223 | 0.000278803 | 0.000286018 |
| Myrmecridium_schulzeri | 0.000227982 | 0.000108699 | 0 | 0 | 0.000316571 |
| Acremonium_alternatum | 6.59E-05 | 0.000166213 | 0.000547487 | 0.000216071 | 0.000336569 |
| Stephanosporaceae_sp | 0 | 1.27E-05 | 0.000134504 | 0.000216611 | 0.000387898 |
| Chrysosporium_pilosum | 0 | 0 | 8.89E-05 | 2.52E-05 | 0.000450102 |
| Kondoa_sorbi | 0 | 0 | 5.46E-06 | 1.61E-05 | 0.000455597 |
| Coniochaetales_sp | 0 | 0 | 0.000103249 | 9.15E-05 | 0.000489983 |
| Tomentella_sp | 4.22E-05 | 0.000121029 | 0.000422428 | 0.000830875 | 0.00053569 |
| Stachybotrys_globosa | 0 | 5.59E-05 | 0 | 8.84E-06 | 0.000721434 |
| Trichoderma_stromaticum | 0.000312946 | 0.000199874 | 9.61E-07 | 2.45E-05 | 0.000726786 |
| Penicillium_sp | 0.000129513 | 0.000133219 | 0.00031973 | 0.000588252 | 0.000741521 |
| Other | 0 | 6.25E-05 | 0.000122592 | 9.77E-05 | 0.00076533 |
| Solicoccozyma_terrea | 0 | 0 | 7.32E-05 | 0.000153144 | 0.000876741 |
| Pluteus_eludens | 0.000283338 | 0.000235525 | 1.47E-05 | 2.80E-05 | 0.000904708 |
| Diversisporaceae_sp | 0 | 9.71E-06 | 5.06E-05 | 2.01E-05 | 0.000957539 |
| Cortinarius_sp | 5.32E-05 | 0.000310178 | 0.0015036 | 0.002080773 | 0.000990547 |
| Pseudaleuria_sp | 0.001795271 | 0.001939901 | 0.021773811 | 0.028537555 | 0.00101592 |
| Solicoccozyma_terricola | 0 | 0 | 8.59E-05 | 7.06E-05 | 0.001027676 |
| Lophiostoma_sp | 0 | 0 | 5.14E-05 | 2.24E-05 | 0.001101638 |
| Clonostachys_sp | 0.000169617 | 0.000110982 | 0.000474496 | 0.00027591 | 0.0011198 |
| Colletotrichum_truncatum | 0 | 0 | 3.52E-05 | 0 | 0.001143074 |
| Modicella_reniformis | 0 | 0 | 2.79E-05 | 0 | 0.001143074 |
| Trichocladium_opacum | 0 | 0 | 2.58E-05 | 0 | 0.001143074 |
| Cristinia_sp | 0.000279791 | 0.000159926 | 0 | 0 | 0.001179565 |
| Auriculariales_sp | 0.00019711 | 0.000635024 | 0.005062224 | 0.00591688 | 0.001241379 |
| Podospora_araneosa | 9.80E-05 | 6.83E-05 | 0.000247959 | 0.000419975 | 0.001291919 |
| Apodus_deciduus | 0 | 7.31E-05 | 0.000230911 | 9.65E-05 | 0.001656484 |
| Coprinellus_sp | 0 | 8.02E-05 | 0.000164994 | 0.000172947 | 0.001665135 |
| Cladosporium_sp | 0 | 1.80E-05 | 4.92E-05 | 8.04E-05 | 0.001940682 |
| Coprinopsis_erythrocephala | 0.000226812 | 6.32E-05 | 0 | 0 | 0.001962326 |
| Hannaella_oryzae | 0 | 0 | 5.68E-05 | 8.19E-05 | 0.002219521 |
| Clavaria_sp | 0.000247261 | 8.49E-05 | 0 | 0 | 0.002255044 |
| Penicillium_oxalicum | 0 | 0.000133661 | 0.00013245 | 3.25E-05 | 0.002311644 |
| Mortierellales_sp | 0.002991573 | 0.00285129 | 0.006386585 | 0.003701526 | 0.002464254 |
| Psilocybe_inquilina | 0.000354025 | 0.00034639 | 3.23E-05 | 6.64E-05 | 0.002701018 |
| Clavicipitaceae_sp | 0.00011539 | 8.79E-05 | 0 | 0 | 0.002709653 |
| Helotiaceae_sp | 0.000113548 | 0.000117118 | 0 | 0 | 0.002903446 |
| Eremiomyces_echinulatus | 0.000111026 | 0.000142334 | 0 | 0 | 0.00294047 |
| Coprinellus_bisporus | 4.09E-05 | 4.27E-05 | 0.000371132 | 0.000127545 | 0.003233082 |
| Arthrographis_kalrae | 0 | 4.08E-05 | 0.000170651 | 5.41E-05 | 0.003295531 |
| Glomus_sp | 0.000139179 | 0.00024335 | 1.39E-05 | 1.11E-05 | 0.003309113 |
| Chaetomium_sp | 0.001141638 | 0.001090992 | 0.00049725 | 0.000940432 | 0.003366814 |
| Endosporium_aviarium | 0 | 0 | 9.20E-06 | 2.81E-05 | 0.003416477 |
| Trichoderma_asperellum | 0.002801559 | 0.002891863 | 0.001550981 | 0.00245546 | 0.003596844 |
| Blumeria_graminis | 0 | 1.58E-05 | 4.66E-05 | 1.04E-05 | 0.003714768 |
| Clitopilus_sp | 9.31E-05 | 0.000237538 | 0 | 0 | 0.003764424 |
| Mortierella_umbellata | 0 | 0 | 2.25E-05 | 3.76E-05 | 0.004426456 |
| Mycosphaerellaceae_sp | 0.000136808 | 0.000274116 | 0.000835219 | 0.000648691 | 0.004447719 |
| Trichocladium_pyriforme | 0 | 2.02E-05 | 5.51E-05 | 9.54E-06 | 0.004989434 |
| Pisolithus_orientalis | 9.68E-05 | 7.56E-05 | 0.00053869 | 0.000835449 | 0.00516994 |
| Polyporales_sp | 0 | 1.45E-05 | 0.000131357 | 0.000101472 | 0.005241166 |
| Pezizaceae_sp | 0.000301004 | 0.000420062 | 7.30E-05 | 4.66E-05 | 0.005418082 |
| Arachnomyces_gracilis | 0 | 0 | 2.59E-05 | 7.95E-07 | 0.005496544 |
| Rhizophydiales_sp | 4.69E-05 | 5.62E-05 | 0.00044622 | 0.000106164 | 0.005622472 |
| Cyphellophora_suttonii | 0 | 0 | 4.77E-05 | 0 | 0.005629315 |
| Mortierella_ambigua | 0 | 0 | 4.17E-05 | 0 | 0.005629315 |
| Boubovia_sp | 0 | 0 | 3.41E-05 | 0 | 0.005629315 |
| Mortierella_capitata | 0 | 0 | 2.37E-05 | 0 | 0.005629315 |
| Graphostroma_platystoma | 0 | 0 | 1.92E-05 | 0 | 0.005629315 |
| Monographella_nivalis | 0 | 0 | 1.11E-05 | 0 | 0.005629315 |
| Aspergillus_deflectus | 0 | 0 | 1.01E-05 | 0 | 0.005629315 |
| Chaetosphaeria_chloroconia | 0 | 0 | 2.86E-05 | 0.000115374 | 0.005791696 |
| Ochroconis_globalis | 0 | 3.81E-06 | 4.69E-05 | 6.43E-06 | 0.005938197 |
| Melanophyllum_haematospermum | 5.77E-05 | 0 | 0 | 0 | 0.006085219 |
| Marasmiellus_tricolor | 0.000213179 | 0.000304484 | 2.71E-05 | 1.10E-05 | 0.007002387 |
| Geomyces_sp | 0 | 1.05E-05 | 4.11E-05 | 1.61E-06 | 0.00703299 |
| Archaeospora_sp | 0 | 0 | 2.26E-05 | 7.02E-06 | 0.007348015 |
| Trechisporales_sp | 0 | 0 | 6.89E-05 | 9.34E-05 | 0.007591152 |
| Ascobolus_sp | 0 | 2.22E-06 | 5.30E-05 | 6.97E-05 | 0.007766927 |
| Entoloma_korhonenii | 0 | 0 | 2.20E-05 | 4.78E-06 | 0.007808162 |
| Chaetomium_erectum | 0.000148325 | 5.37E-05 | 0.000100159 | 4.26E-05 | 0.007942817 |
| Inocybe_rimosa | 0 | 8.84E-05 | 0 | 0 | 0.008402918 |
| Trametes_versicolor | 0 | 0 | 2.70E-05 | 1.83E-05 | 0.008496834 |
| Exophiala_sp | 0 | 2.93E-05 | 0.000106109 | 6.15E-05 | 0.009249456 |
| Paramyrothecium_roridum | 0 | 0 | 2.80E-05 | 3.29E-05 | 0.0094538 |
| Stachybotrys_echinata | 0 | 0.000132531 | 0.000108282 | 8.49E-05 | 0.009882332 |
| Oidiodendron_truncatum | 7.51E-05 | 0 | 4.85E-05 | 9.35E-05 | 0.01007541 |
| Chaetothyriales_sp | 7.20E-05 | 0.00028763 | 0.000409136 | 0.000353873 | 0.010572678 |
| Fusarium_delphinoides | 4.72E-05 | 6.86E-06 | 0.000119183 | 7.71E-05 | 0.010975361 |
| Apiotrichum_veenhuisii | 2.58E-05 | 3.99E-05 | 6.63E-05 | 0.000150891 | 0.011375638 |
| Limnoperdon_incarnatum | 0.000130075 | 0.000162187 | 0 | 0 | 0.011440609 |
| Monosporascus_sp | 7.03E-05 | 2.66E-05 | 7.51E-05 | 2.92E-05 | 0.011521744 |
| Xylariales_fam_Incertae_sedis_sp | 3.77E-05 | 0 | 3.60E-05 | 2.41E-05 | 0.011724127 |
| Plantae_sp | 0.000117277 | 0.000193358 | 1.58E-05 | 0 | 0.012558163 |
| Phialophora_hyalina | 0 | 0 | 5.75E-05 | 6.94E-05 | 0.01273086 |
| Trapelia_coarctata | 0 | 0 | 3.29E-05 | 5.21E-05 | 0.013410613 |
| Phaeoacremonium_hungaricum | 2.90E-05 | 0.000176475 | 0.000249854 | 0.000141065 | 0.014658244 |
| Cordyceps_sp | 0 | 0 | 8.90E-05 | 1.84E-05 | 0.015449466 |
| Filobasidiales_sp | 0 | 0 | 1.91E-05 | 8.59E-05 | 0.015811438 |
| Ascomycota_sp | 0.016244862 | 0.018983508 | 0.033636917 | 0.022935173 | 0.016257604 |
| Thielavia_arenaria | 1.87E-05 | 0 | 9.19E-06 | 0.000184313 | 0.016307469 |
| Acremonium_curvulum | 0 | 0 | 4.10E-05 | 8.89E-06 | 0.01642735 |
| Lectera_longa | 0.000167841 | 6.35E-05 | 0.000176167 | 0.00087671 | 0.016705716 |
| Penicillium_levitum | 0.002413934 | 0.002404546 | 0.001480828 | 0.002663769 | 0.017088559 |
| Trichoderma_turrialbense | 0 | 0 | 3.03E-05 | 1.77E-05 | 0.017447957 |
| Bolbitiaceae_sp | 0 | 0 | 3.31E-05 | 1.12E-05 | 0.017447957 |
| Aspergillus_fischeri | 0.000109902 | 0.000125315 | 0.000292524 | 0.000229115 | 0.017545537 |
| Candida_parapsilosis | 0.000144354 | 2.11E-05 | 5.99E-05 | 0.000161177 | 0.01838319 |
| Lentitheciaceae_sp | 0 | 0 | 2.24E-05 | 7.18E-06 | 0.018511664 |
| Laetisaria_fuciformis | 0 | 0 | 7.53E-05 | 7.26E-05 | 0.019573644 |
| Typhula_sp | 0 | 0 | 2.97E-05 | 2.05E-05 | 0.019618732 |
| Pleosporaceae_sp | 0.002198173 | 0.0022985 | 0.003944015 | 0.00250881 | 0.019848518 |
| Candida_cretensis | 0 | 0 | 8.63E-05 | 2.66E-05 | 0.020402213 |
| Picoa_juniperi | 1.72E-06 | 5.74E-05 | 0.000112333 | 0.000145961 | 0.021190862 |
| Ceratobasidiaceae_sp | 0.000451674 | 0.000683972 | 0.001047729 | 0.000853762 | 0.021340867 |
| Acremonium_dichromosporum | 0.000357225 | 0.00048169 | 0.000604228 | 0.000241332 | 0.02160328 |
| Chytridiomycota_sp | 0.000127712 | 0.000259721 | 0.000552079 | 0.000523737 | 0.022227363 |
| Chytridiomycetes_sp | 6.54E-05 | 6.34E-05 | 0.001904511 | 0.000201427 | 0.02344582 |
| Ascobolaceae_sp | 1.04E-05 | 5.25E-05 | 0 | 0 | 0.023632306 |
| Tremellales_fam_Incertae_sedis_sp | 0.00010709 | 0.00015558 | 0.00033909 | 0.000161202 | 0.023703776 |
| Custingophora_olivacea | 3.69E-05 | 0.000230153 | 0.000379877 | 0.000387757 | 0.024131723 |
| Acremonium_vitellinum | 7.67E-05 | 0 | 4.97E-05 | 3.17E-05 | 0.024333872 |
| Hebeloma_cavipes | 2.44E-05 | 9.06E-05 | 0 | 0 | 0.024661229 |
| Helotiales_sp | 0.001284074 | 0.000979475 | 0.001328082 | 0.000600712 | 0.024668195 |
| Anguillospora_longissima | 0 | 0 | 2.10E-05 | 8.07E-07 | 0.024736596 |
| Clavaria_acuta | 0.000109831 | 3.51E-05 | 0 | 0 | 0.025551949 |
| Peziza_sp | 0.000267167 | 8.96E-05 | 0 | 0 | 0.025589432 |
| Ophiosphaerella_agrostidis | 0 | 0 | 2.54E-05 | 0 | 0.025688375 |
| Sporormiella_pulchella | 0 | 0 | 2.35E-05 | 0 | 0.025688375 |
| Powellomyces_sp | 0 | 0 | 2.21E-05 | 0 | 0.025688375 |
| Preussia_longisporopsis | 0 | 0 | 1.83E-05 | 0 | 0.025688375 |
| Phaeoacremonium_rubrigenum | 0 | 0 | 1.69E-05 | 0 | 0.025688375 |
| Erythricium_atropatanum | 0 | 0 | 1.46E-05 | 0 | 0.025688375 |
| Russula_rosea | 0 | 0 | 8.45E-06 | 0 | 0.025688375 |
| Stilbella_sp | 0 | 0 | 8.33E-06 | 0 | 0.025688375 |
| Coprinopsis_narcotica | 0 | 0 | 6.40E-06 | 0 | 0.025688375 |
| Funneliformis_sp | 0 | 0 | 5.59E-06 | 0 | 0.025688375 |
| Gibberella_sp | 0 | 0 | 3.65E-06 | 0 | 0.025688375 |
| Stagonospora_perfecta | 2.35E-05 | 8.98E-05 | 0 | 0 | 0.025855741 |
| Aureobasidium_pullulans | 0 | 0 | 2.24E-05 | 4.78E-06 | 0.02602428 |
| Hirsutella_vermicola | 0 | 0.000100858 | 0 | 0 | 0.026408369 |
| Pyrenochaeta_sp | 0 | 0 | 2.10E-05 | 7.97E-07 | 0.026761012 |
| Geoglossaceae_sp | 3.52E-05 | 8.57E-05 | 0 | 0 | 0.027062163 |
| Hannaella_siamensis | 4.09E-05 | 2.05E-05 | 3.71E-06 | 2.06E-05 | 0.027268312 |
| Agaricales_sp | 0.00289108 | 0.002507095 | 0.003998505 | 0.00363787 | 0.02742247 |
| Lecanicillium_kalimantanense | 8.54E-05 | 0.00017685 | 0 | 0 | 0.028276971 |
| Didymosphaeria_sp | 3.35E-06 | 1.21E-05 | 0 | 0 | 0.028276971 |
| Coprinellus_brevisetulosus | 2.01E-05 | 8.73E-05 | 0.000153721 | 0.000113145 | 0.029188212 |
| Spizellomyces_pseudodichotomus | 0 | 0 | 0.000204437 | 4.76E-06 | 0.03117837 |
| Ophiocordycipitaceae_sp | 0 | 2.31E-05 | 1.65E-05 | 0 | 0.031518754 |
| Pyrenochaetopsis_leptospora | 0.000833013 | 0.000891681 | 0.000675344 | 0.000365692 | 0.031524941 |
| Microbotryomycetes_sp | 0 | 0 | 4.15E-05 | 4.35E-05 | 0.031692569 |
| Ophiostomatales_sp | 0 | 0 | 5.24E-05 | 0.000114968 | 0.033577174 |
| Peziza_ammophila | 0 | 7.70E-06 | 1.40E-05 | 0 | 0.034198461 |
| Myxocephala_albida | 9.04E-05 | 0.000139399 | 0.000198886 | 0.000236989 | 0.034202257 |
| Conlarium_dupliciascosporum | 0 | 0 | 1.13E-05 | 7.09E-05 | 0.035712593 |
| Cyphellophora_sp | 0.000102741 | 9.96E-05 | 9.19E-05 | 5.57E-06 | 0.036123066 |
| Cylindrocladiella_sp | 0 | 0 | 2.07E-05 | 4.55E-05 | 0.036437709 |
| Lecanicillium_primulinum | 0 | 1.68E-05 | 3.71E-06 | 3.02E-05 | 0.037307339 |
| Podospora_pyriformis | 2.80E-05 | 4.93E-05 | 7.85E-05 | 0.000119011 | 0.037676033 |
| Eurotiomycetes_sp | 3.41E-06 | 0.000131201 | 8.72E-05 | 8.32E-05 | 0.037862747 |
| Podospora_sp | 0.00072862 | 0.000850101 | 0.000411661 | 0.000479676 | 0.039711836 |
| Melanocarpus_thermophilus | 0.000156787 | 1.53E-05 | 0.000116288 | 0.000105252 | 0.040189497 |
| Erythrobasidiales_sp | 5.78E-05 | 0 | 3.85E-06 | 0 | 0.040400654 |
| Pleosporales_fam_Incertae_sedis_sp | 0.000148049 | 0.000190874 | 0.000166738 | 0.000347216 | 0.04399197 |
| Leptoxyphium_madagascariense | 0.000187598 | 5.33E-06 | 0 | 0 | 0.045380087 |
| Yamadazyma_philogaea | 0 | 0 | 3.92E-05 | 5.22E-05 | 0.047602657 |
| Leptosphaeriaceae_sp | 0 | 0 | 1.94E-05 | 3.26E-05 | 0.048528889 |
| Cystolepiota_cystophora | 6.95E-05 | 0.000117824 | 0 | 0 | 0.049334399 |
| Leucoagaricus_sp | 1.48E-05 | 5.93E-05 | 0 | 0 | 0.049407911 |
| Periconia_macrospinosa | 1.69E-05 | 5.38E-05 | 2.21E-05 | 4.00E-05 | 0.049410487 |

**Supplementary Figures**


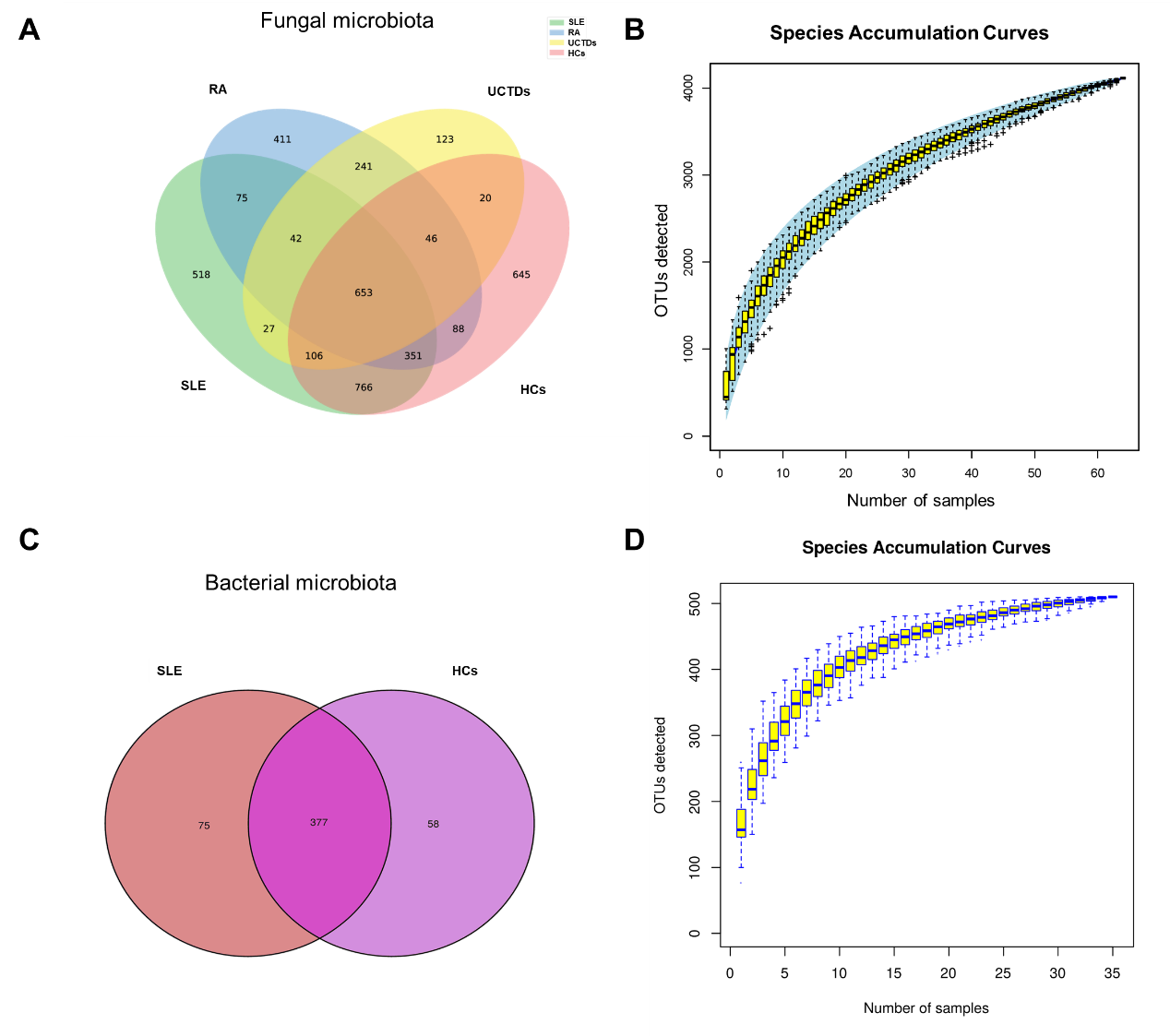


**Supplementary Figure 1.** (**A**) Venn plots of OTUs in groups of ITS sequencing; (**B**) Species accumulation curves of ITS sequencing; (**C**) Venn plots of OTUs in groups of 16S rRNA; (**D**) Species accumulation curves of 16S rRNA.


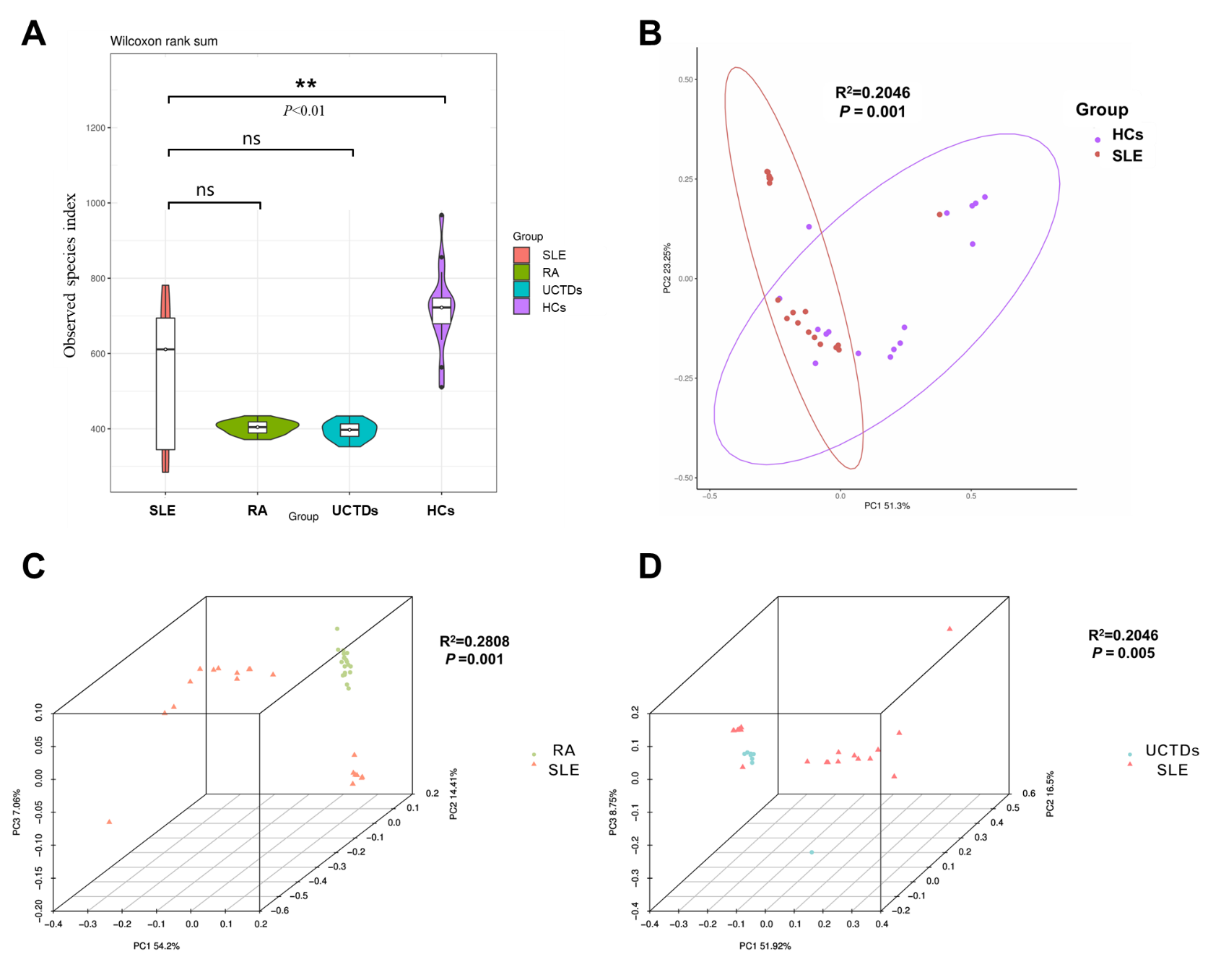


**Supplementary Figure 2.** Alpha and beta Diversities of gut fungi between SLE, RA, UCTDs and HCs

(**A**) Alpha diversities with observed species for SLE compared with HCs, RA and UCTDs; (**B**) Principal coordinate analysis (PCoA) on Bray-Curtis distance of the fungal microbiota community structures in SLE and HCs; (**C**) Principal coordinate analysis (PCoA) on Bray-Curtis distance of the fungal microbiota community structures in SLE and RA; (**D**) Principal coordinate analysis (PCoA) on Bray-Curtis distance of the fungal microbiota community structures in SLE and UCTDs.


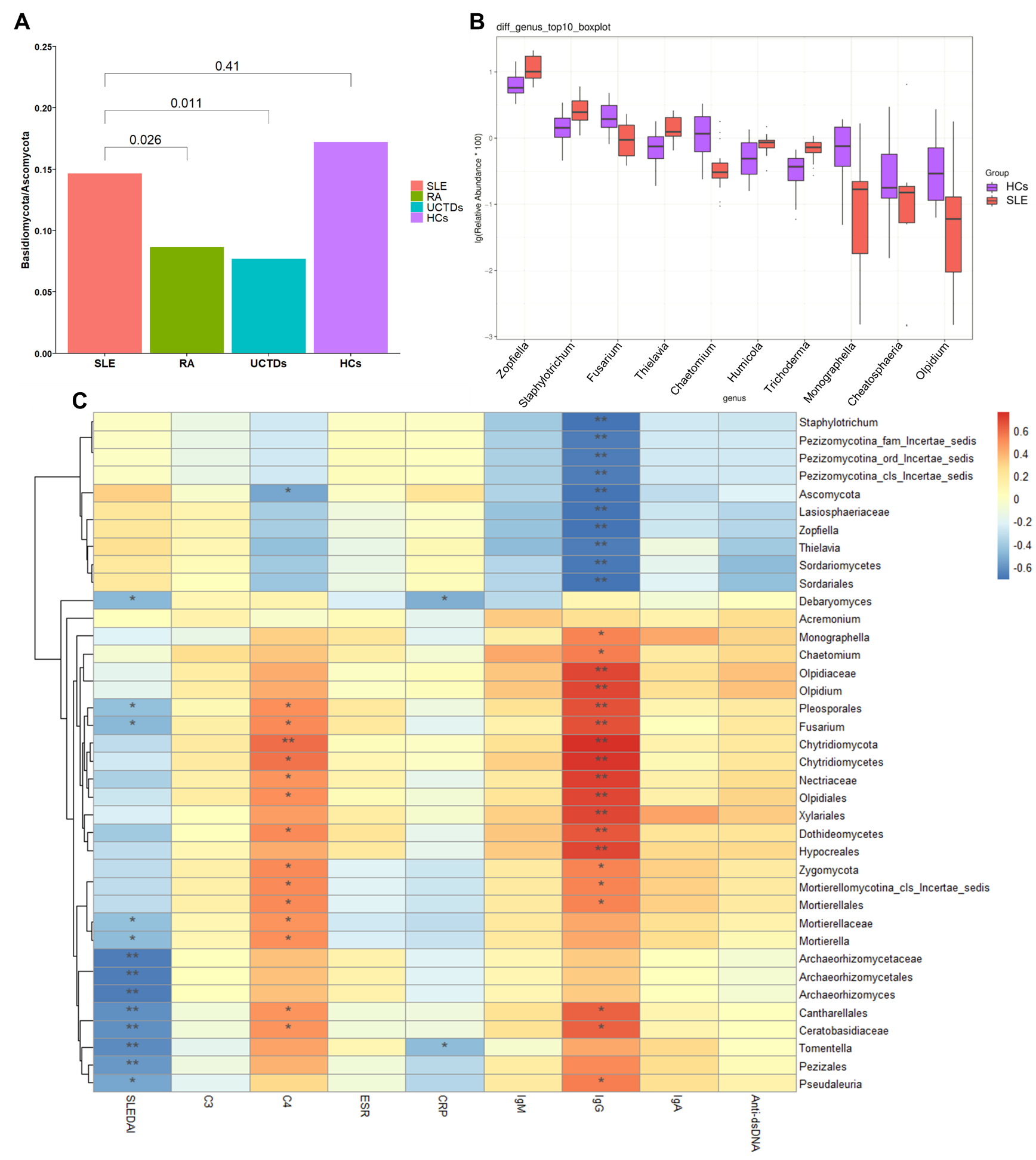
**Supplementary Figure 3.** (A)The average ratios of Basidiomycota/Ascomycota; (B)The top ten fungi with significant differences at genus level between SLE and HCs; (C) Association between differential fungi and clinical indicators.


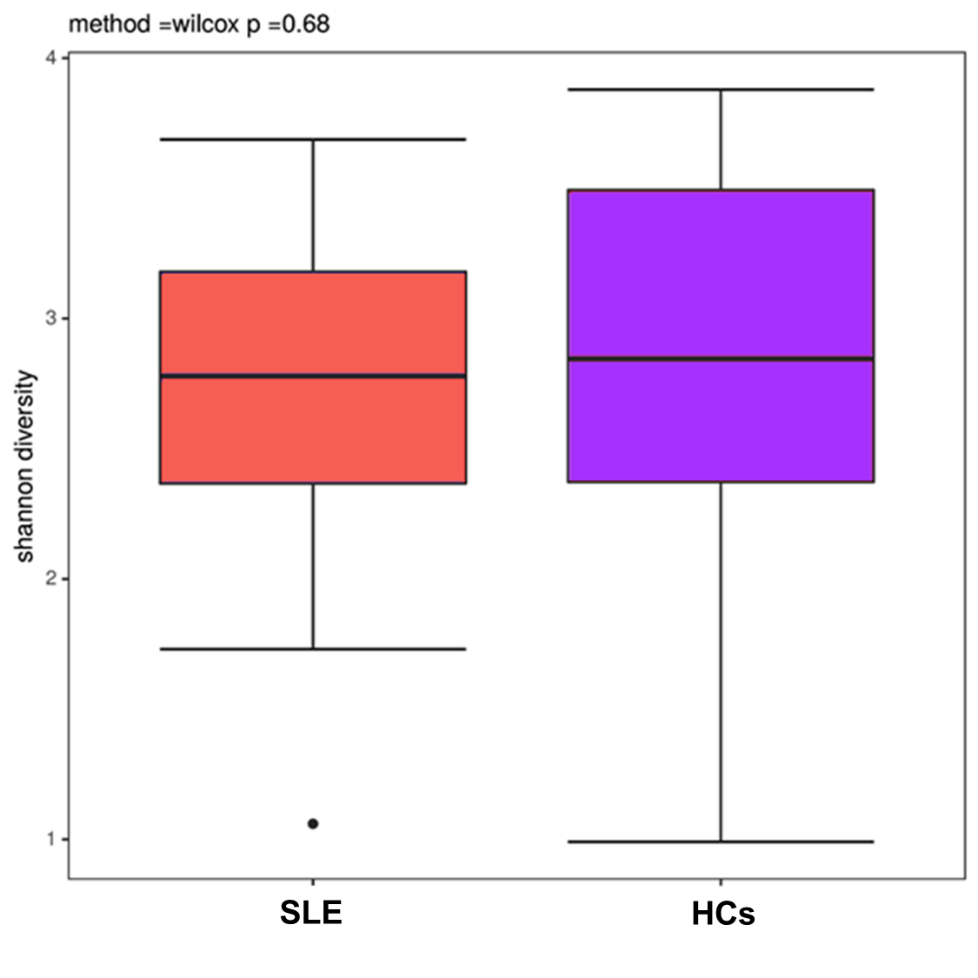


**Supplementary Figure 4.** The alpha diversity with Shannon index of bacterial microbiota community between SLE and HCs.


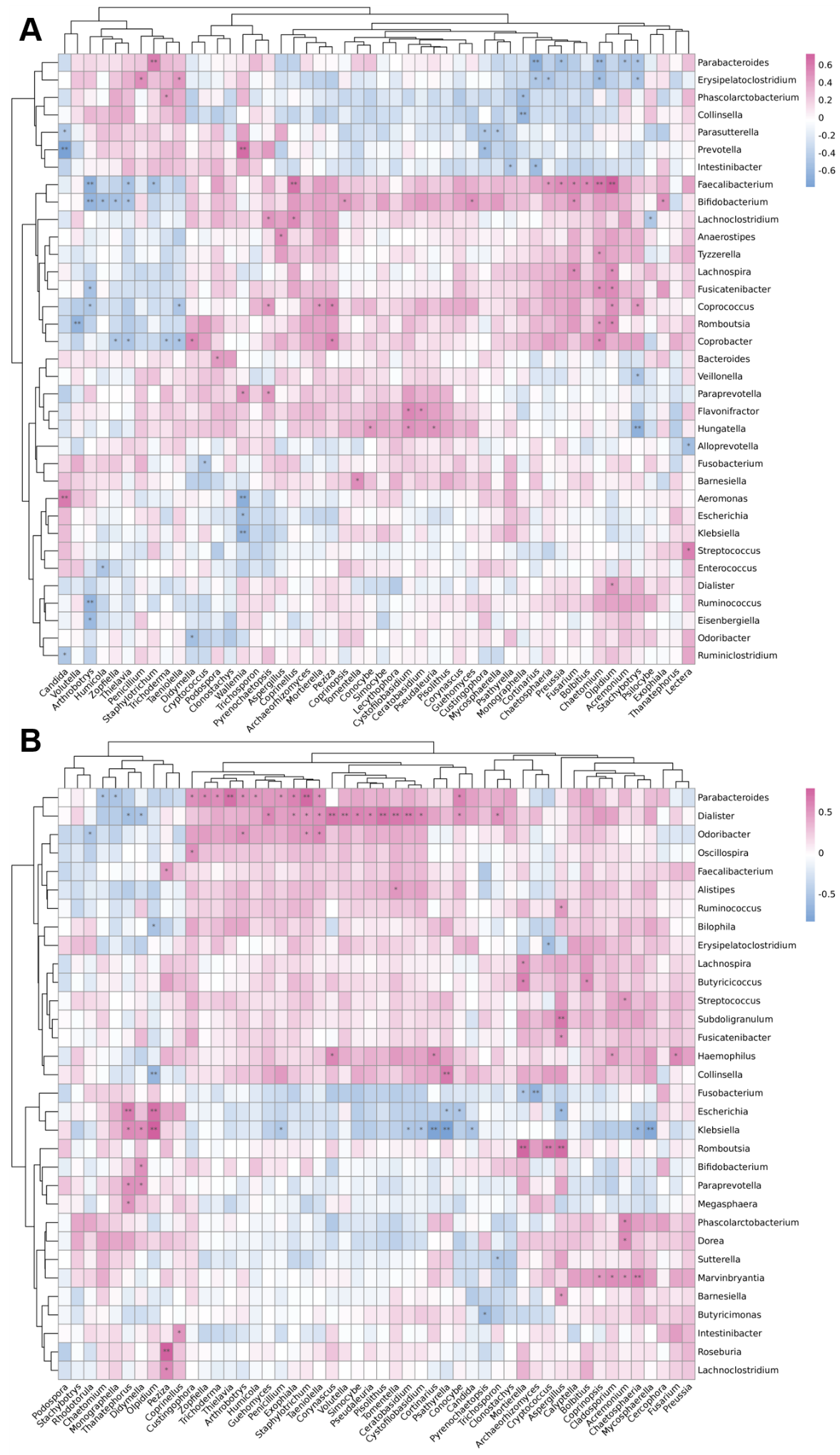
**Supplementary Figure 5** The correlation between bacterial and fungal microbiota is shown in SLE (**A**) and HCs (**B**).
